# A revision of *Lachnodius* Maskell (Hemiptera, Coccomorpha, Eriococcidae)

**DOI:** 10.3897/zookeys.818.32061

**Published:** 2019-01-21

**Authors:** Nate B. Hardy, John W. Beardsley Jr, Penny J. Gullan

**Affiliations:** 1 Department of Entomology and Plant Pathology, Auburn University, Auburn, Alabama, USA Auburn University Auburn United States of America; 2 Formerly of Department of Entomology, University of Hawaii, Hawaii, U.S.A.; deceased 5 February 2001 University of Hawaii Hawaii United States of America; 3 Division of Ecology & Evolution, Research School of Biology, The Australian National University, Acton, A.C.T., 2601, Australia Australian National University Canberra Australia

**Keywords:** Australian endemics, gall-inducer, taxonomy

## Abstract

*Lachnodius* Maskell is a genus of three named species that are part of an Australian radiation of felt scale insects that induce galls on *Eucalyptus* and *Corymbia* (Myrtaceae). A female’s gall usually consists of an open-top pit in swollen plant tissue. Depending on the species, galls can occur on a host’s leaves, buds, stems, or trunk. Here, we redescribe the named species: *L.eucalypti* (Maskell), *L.hirsutus* (Froggatt) and *L.lectularius* (Maskell), and describe seven new species: *L.brimblecombei* Beardsley, Gullan & Hardy, **sp. n.**, *L.froggatti* Beardsley, Gullan & Hardy, **sp. n.**, *L.maculosus* Beardsley, Gullan & Hardy, **sp. n**., *L.melliodorae* Beardsley, Gullan & Hardy, **sp. n.**, *L.newi* Beardsley, Gullan & Hardy, **sp. n.**, *L.parathrix* Beardsley, Gullan & Hardy, **sp. n.**, *L.sealakeensis* Gullan & Hardy, **sp. n.** Descriptions are based primarily on adult females, but for some species short diagnoses of nymphal stages also are provided. The taxonomic history of *Lachnodius* is reviewed, with notes on their biology and ecology. A key to species based on the morphology of adult females is provided, and lectotypes are designated for *Dactylopiuseucalypti* Maskell and *Lachnodiuslectularius* Maskell.

## Introduction

In Australia, species of *Eucalyptus* and the closely related genus *Corymbia* are host to many species of gall-inducing felt scale insects (Gullan et al. 2005; García Morales et al. 2016). Most belong to one of two major radiations: the genus *Apiomorpha* Rübsaamen and a distantly related clade of Myrtaceae-feeding species (Cook and Gullan 2004). *Lachnodius* Maskell is one of at least eight genera belonging to the latter clade. Our aim here is to revise *Lachnodius*, with re-descriptions of the three currently recognized species and descriptions of seven new species.

### Taxonomic history and phylogenetic relationships of *Lachnodius*

Maskell (1896) erected the genus *Lachnodius* for *Dactylopiuseucalypti* Maskell, which he had described in 1892, and two other species, *L.hirtus* Maskell and *L.lectularius* Maskell, which he described as new. Fernald (1903) designated *L.eucalypti* as the type species. Beardsley (1982) synonymized the monotypic genus *Pseudopsylla* Froggatt with *Lachnodius* after study of the type specimens of the type species *P.hirsutus* Froggatt. This brought the number of described species of *Lachnodius* to four. Hardy et al. (2011) then transferred *L.hirtus* to their genus *Lobimargo* Hardy & Gullan, and the tally of *Lachnodius* species went back to three. Adult females of *Lachnodius* can be distinguished from other genera of felt scales found on *Eucalyptus* based on the morphological features in the keys of Hardy and Gullan (2007) or Hardy et al. (2011).

In Maskell’s brief definition of *Lachnodius*, he did not speculate on how it was related to other scale insects. Fernald (1903) placed *Lachnodius* in her Dactylopiinae, which included the presently recognized families Asterolecaniidae, Eriococcidae, Kermesidae and Pseudococcidae. Froggatt (1921) and Morrison and Morrison (1922) followed the classification of Fernald. The Morrisons also proposed a close relationship between *Lachnodius* and *Sphaerococcopsis* Cockerell. Ferris (1955) was puzzled by *Lachnodius*; he considered erecting an entirely new family for it, before opting to place it awkwardly in Pseudococcidae. Incidentally, the form that Ferris illustrated under the name *Lachnodiuseucalypti* is certainly not that species, but may be *L.lectularius*. Hoy (1963) assigned both *Lachnodius* and *Sphaerococcopsis* to the Eriococcidae, a family to which he applied broad limits. Beardsley was of the opinion that *Lachnodius* and *Sphaerococcopsis* could not be placed easily into either Eriococcidae or Pseudococcidae (Beardsley 1972, 1974). He agreed with Ferris, that these genera constituted a previously unrecognized family-level taxon, but one that was more closely related to the Eriococcidae than the Pseudococcidae. Koteja (1974) followed suite, and held *Lachnodius* to be a distinct family-level taxon, tentatively placed in his asterolecaniid group of families, on the basis of comparative studies of the labium, salivary pump and clypeolabral shield of adult females. In sum, the phylogenetic relationships of *Lachnodius* and *Sphaerococcopsis* were an enigma.

In the first scale insect phylogeny inferred from DNA sequence data, Cook et al. (2002) found support for a monophyletic group comprised of an unidentified *Lachnodius* species, *Tanyscelismammularis* (Froggatt) and *Ascelispraemollis* Schrader (both of the latter being members of Eriococcidae in its current form); all three species induce galls on myrtaceous hosts. Then, in a more comprehensive estimate of the phylogeny of eriococcids, Cook and Gullan (2004) found these same three taxa inside a clade of Myrtaceae-feeding species that formed part of a larger clade of species from the Southern Hemisphere. They also found that the Eriococcidae is not monophyletic, as per previous suggestions based on morphological studies (Cox and Williams 1987; Hodgson 2002). The classification of scale insects has yet to be reconciled with this finding, but the most likely resolution will entail the recognition of the Myrtaceae-feeding (MF) clade of Cook and Gullan (2004) as a formal family-level taxon. This group would include many other mostly gall-inducing genera in addition to *Lachnodius* and *Sphaerococopsis*. Thus, it seems that Ferris, Beardsley, and Koteja were correct: *Lachnodius* and *Sphaerococcopsis* are not a natural fit in any of the existing scale insect families.

### Undescribed species diversity of Myrtaceae-feeding clade

The MF clade is species rich and divided into subradiations, each of which is largely restricted to a subclade of Myrtaceae (Cook and Gullan 2004; Gullan et al. 2005). The species diversity of radiations on *Leptospermum* and *Melaleuca* is almost entirely undescribed (LG Cook pers. comm.). More progress has been made in documenting the species that feed on *Eucalyptus* and *Corymbia*. In fact, over the last decade we (e.g., Hardy and Gullan 2007, 2010; Hardy et al. 2011; Semple et al. 2015) have approached complete coverage of the known diversity (which, of course, says nothing about the unknown diversity). Here, we make another step in that direction by describing seven new species and redescribing the three already named species of *Lachnodius*.

## Materials and methods

Adult females and immature specimens from recent collections and from dry museum material were slide-mounted in Canada balsam, mainly using a method similar to that described in Gullan (1984b). The morphological terms mainly follow Williams (1985), Miller and McKenzie (1967) and Hardy and Gullan (2007). The adult females of a few species have tiny dorsal sclerotic pits or depressions that are referred to herein as urns or varioles, depending on their shape. Measurements were made using an ocular micrometer attached to a compound microscope. All are given as a range and based on maximum dimensions (e.g., the body width of a slide mounted specimen was measured across the widest transverse section, the location of which varies among specimens, and leg segment lengths were measured along the longest axis). Tarsal length excludes the claw. Spiracle length includes the muscle plate (apodeme). Setal lengths exclude the setal base. All illustrations of the insects were prepared by NBH and photographs of the live insects and galls were taken by PJG.

Depositories are abbreviated as follows:

**ANIC**Australian National Insect Collection, CSIRO, Canberra, ACT, Australia;

**ASCU**Agricultural Scientific Collections Unit, New South Wales Department of Primary Industries, Orange Agricultural Institute, Orange, New South Wales, Australia;

**NHMUK**The Natural History Museum, London;

**BPBM**Bernice P Bishop Museum, Honolulu, Hawaii, USA;

**NMV**Museum of Victoria, Melbourne, Australia;

**NZAC**New Zealand Arthropod Collection, Landcare Research, Auckland, New Zealand;

**QDPC**Department of Primary Industries Insect Collection, Brisbane, Queensland, Australia;

**QM**Queensland Museum, Brisbane, Queensland, Australia;

**WAM**Western Australian Museum, Perth, Western Australia, Australia;

**USNM** the United States National Collection of Coccoidea of the National Museum of Natural History, Smithsonian Institution, housed at the United States Department of Agriculture, Beltsville, Maryland, USA.

The NZAC houses original slides and dry material of species described by WM Maskell and follows the principle that primary type material should reside in the country of origin of the species, if suitable repositories exist (Deitz and Tocker 1980); thus when lectotypes are designated for Maskell specimens collected in Australia, these specimens can be deposited in the ANIC, as we do here.

Beardsley examined Maskell material at NZAC in 1972 and borrowed and slide-mounted specimens from the pill boxes containing Maskell dry material of *Lachnodius*; unfortunately, the original boxes that Beardsley borrowed appear to have been lost after his death as they were not returned to NZAC (RC Henderson, pers. comm.). The late Helen Brookes (formerly at the Waite Agricultural Research Institute, University of Adelaide, South Australia) acquired and curated a large collection of Coccoidea (including *Lachnodius*) during her career and, following her retirement in 1982, deposited this collection and associated notes and photographs in the Australian National Insect Collection (Upton 1997; Taylor and Keller 2008); she also recorded biological and other information on cards (Gullan and Williams 2010) filed by a Specimen Index Number that consisted of a number for the collection and an abbreviation of the year, for example, 31/67 was her 31^st^ collection for 1967. We have included some of her information on the biology and appearance of *Lachnodius*. Some slide-mounted specimens are DNA vouchers of LG Cook and/or NBH and have voucher codes (e.g., LGC01374, NH150); all are deposited in ANIC. Collector and author names are abbreviated as follows: JWB, JW Beardsley; HMB, HM Brookes, NBH, NB Hardy; PJG, PJ Gullan.

The International Commission on Zoological Nomenclature (1999) requires lectotypes designated after 1999 to “contain an express statement of deliberate designation” (amended Article 74.7.3). We use the statement “here designated” to fulfil this requirement. We have registered each of the new names published in this paper with the Official Registry of Zoological Nomenclature (ZooBank) and cite the Life Science Identifiers (LSIDs) after the heading for each new name. Each LSID is a globally unique identifier for the nomenclatural act of naming a new taxon.

JWB is the coauthor of six new names for *Lachnodius* because he recognized these species in his unpublished work. We provide a short synopsis of his work in the Discussion. A large portion of this study was based upon JWB’s collection, which is housed in the BPBM. The BPBM has allowed the holotype of any new Australian species from the JWB collection to be deposited in the ANIC (in correspondence of PJG in 1996).

### Notes on parasitoids and the effects of parasitization

Slide-mounted adult female and second-instar specimens of *Lachnodius* frequently show evidence of attack by internal parasitoids. We have noted the chorion of parasitoid eggs and developing parasitoid larvae, sometimes evident only by their mandibles. In the field, JWB occasionally found obviously parasitized adult females of both *L.eucalypti* and *L.lectularius* within their galls on host trees. As the parasitoids matured, the parasitized host became a hard, brown husk from which the adult wasps eventually emerged. Several parasitoids can develop in one host, with as many as 20 adults emerging from a single adult female of *L.lectularius*. JWB determined that the wasps were a kind of Encyrtidae, possibly species of *Metaphycus* or related genera. There are no previously published records of parasitoids attacking *Lachnodius*.

It appears that parasitization affects the development of structures in the host’s integument, in particular the macrotubular ducts, making identification of parasitized specimens potentially problematic. Compared to unparasitized individuals, parasitized female specimens identified as *L.lectularius* often have fewer or smaller macrotubular ducts. Normally, the dorsal macrotubular ducts are numerous and large (ca. 6–8 µm rim diameter). In some parasitized specimens the ducts are few, whereas in others they are abundant but small (ca. 2–3 µm in diameter and without well-defined rims).

## Taxonomy

### Lachnodius

Taxon classificationAnimaliaHemipteraEriococcidae

Maskell, 1896

Lachnodius Maskell, 1896: 400. Type species: *Dactylopiuseucalypti* Maskell. Subsequently designated by Fernald 1903.
Pseudopsylla : Froggatt 1921: 6. Type species: *Pseudopsyllahirsutus* Froggatt, by monotypy and original designation. Synonymy by Beardsley 1982: 31.

#### Biological notes.

**Figure 1.**
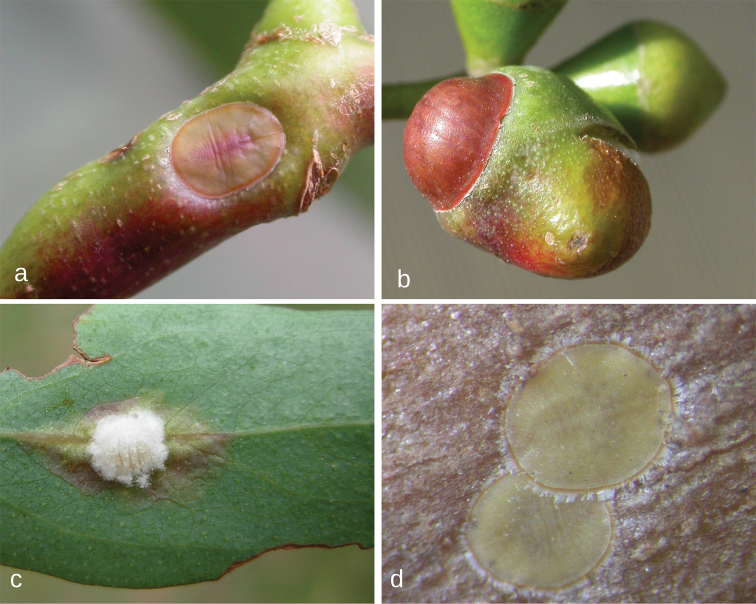
Species of *Lachnodius* in life: **a** gall of *L.brimblecombei* on stem of *Eucalyptusbaxteri*, Grampians, Victoria **b** leaf discoloration surrounding pit galls induced by *L.eucalypti* on *E.blakelyi*, near Forbes, New South Wales **c** mature adult females and ovisacs of *L.eucalypti* on trunk of *E.mannifera*, Canberra, A.C.T. **d** adult female of *L.froggatti* in pit gall on leaf of *E.baueriana*, near Narooma, N.S.W. **e** same female of *L.froggatti* removed from its pit gall **f** gall of *L.hirtus* on *Corymbianesophila*, Gunn Point, Northern Territory.

**Figure 2.**
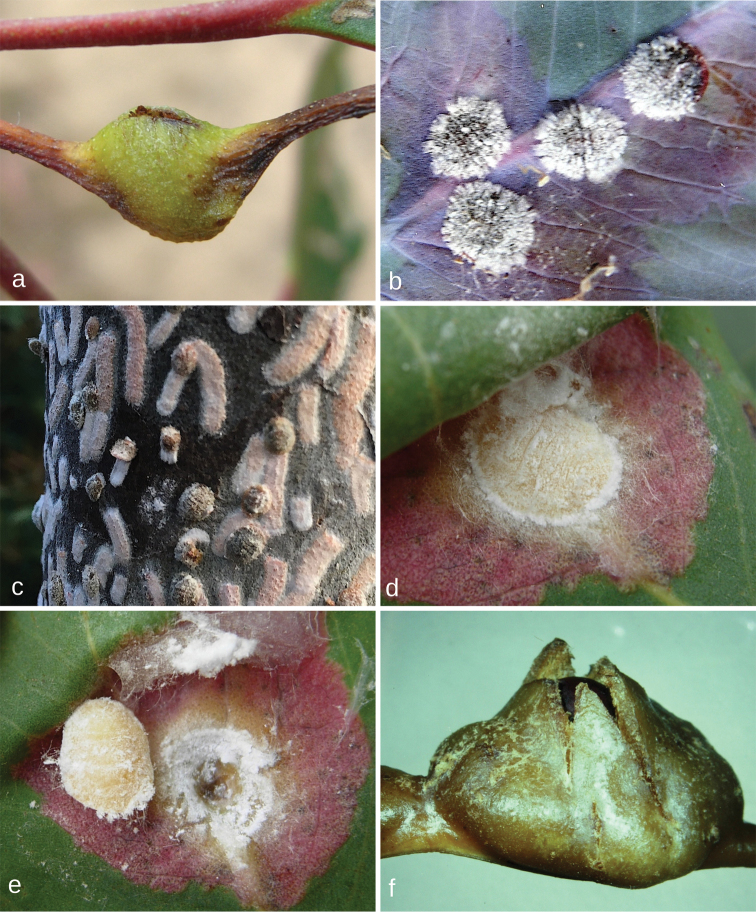
Species of *Lachnodius* in life. **a** adult female of *L.lectularius* in pit gall on stem of *Eucalyptusviminalis*, Cranbourne, Victoria **b** adult female of *L.lectularius* in pit gall on bud of *E.viminalis*, Tyabb, Victoria **c** adult female of *L.parathrix* in pit gall on mid-vein of *E.elata*, near Narooma, New South Wales **d** two adult females of *L.sealakeensis* in pits on trunk of *E. ?oleosa*, near Sea Lake, Victoria.

The females of all species of *Lachnodius* induce galls of varying complexity on the leaves, buds, stems, or main trunk of species of *Eucalyptus* or *Corymbia* (Myrtaceae) (Figs 1, 2). Galls consist of a pit in swollen plant tissue with insect’s dorsum either exposed or partially concealed. Females remain in their gall after their imaginal molt, and then at maturity, depending on species, either desert their gall and move elsewhere for oviposition, or remain in their gall for reproduction. Males, in the few species for which they are known, induce galls as first-instar nymphs but then, near the end of their second instar, vacate the gall and move to another site to form cocoons in which they complete their development.

#### Diagnosis of adult females of the genus *Lachnodius*

Body outline circular to oval. In most species eyes on margin (on venter in *L.froggatti*, and absent in *L.sealakeensis*). Antennae six to seven-segmented. Pair of broad, blister-like frontal lobes between antennae; a series of elongate setae along posterior margin of lobes. Tentorial box usually with anterior aliform extensions. Labium either one-segmented, or composed of two fused segments; proximal segment indicated by a pair of setae on ventral surface; distal segment with one pair of ventral seta, one pair of fleshy apical setae, and one pair of dorsal setae. Legs well developed. Anus ventral, with sclerotic rim having fewer than ten setae (except in *L.hirsutus*), base of each seta surrounded by ring of minute pores. Anal lobes absent.

*Dorsum*. Setae short to minute, ≤ 10 µm long (except up to 25 µm long on *L.hirsutus*). Microtubular ducts and one or two size classes of macrotubular ducts present; larger macrotubular ducts sometimes with one seta touching rim of dermal orifice; duct shaft of uniform width or constricted near vestibule; macrotubular ducts with vestibule weakly sclerotic and compressed, i.e., not cup-shaped. Derm membranous, sometimes with enlarged microtrichia, sometimes with concave sclerotic granules. Multilocular pores absent. Dorsum delimited by a marginal with fringe of setae, differentiated from other body setae, with shape flagellate, conical or sagittate; marginal fringe either complete around margin, or with break between thorax and abdomen, or with break between thorax and abdomen + break between meso- and metathorax.

*Venter*. Sometimes larger than dorsum. Setae flagellate, in transverse rows across each abdominal segment, scattered along submargin, in clusters anterior to each coxa. Microtubular ducts usually absent (*L.eucalypti* with scattered microtubular ducts on head); macrotubular ducts similar to those on dorsum. Quinquelocular pores dense around vulva, clusters around each spiracle, scattered along submargin and across each body segment.

#### Etymology.

Although Maskell (1896) did not explicitly state the meaning of the genus name that he coined, his description included a statement that the female insects were either naked or covered in cottony or mealy or waxy secretion. The name *Lachnodius* thus must be derived from the masculine Greek noun *lachno*, meaning woolly hair or down.

#### Key to species of *Lachnodius* based on adult females

**Table:** 

1	Anal ring set at base of sclerotic invagination	**2**
–	Anal ring flush with body surface, or if recessed not at base of sclerotic invagination	**6**
2	Marginal setae fine, strongly recurved	**3**
–	Marginal setae stout, conical	**4**
3	Dorsum beset with minute, urn-shaped sclerites; some dorsal macrotubular ducts with base of a seta touching rim of dermal orifice	***Lachnodiusmelliodorae* sp. n.**
–	Dorsum beset with enlarged, sclerotic microtrichia; no dorsal macrotubular ducts with base of a seta touching rim of dermal orifice	***Lachnodiusnewi* sp n.**
4	Eyes absent; dorsum with small concave sclerites, each bearing a tubular duct	***Lachnodiussealakeensis* sp. n.**
–	Eyes on margin; dorsum with or without small concave sclerites, if with, then each lacking a tubular duct	**5**
5	Dorsum beset with minute, concave sclerites	***Lachnodiusmaculosus* sp. n.**
–	Dorsum without minute, concave sclerites	***Lachnodiusparathrix* sp. n.**
6	Eyes on ventral surface of head; some dorsal macrotubular ducts with base of a seta touching rim of dermal orifice	***Lachnodiusfroggatti* sp. n.**
–	Eyes on margin; no dorsal macrotubular ducts with base of a seta touching rim of dermal orifice	**7**
7	Marginal fringe of alternating sagittate and slender conical setae, both types of setae short (up to 20 µm long); labium one-segmented; microtubular ducts present on ventral surface of head	***Lachnodiuseucalypti* (Maskell)**
–	Marginal fringe setae conical, or flagellate, long (38–455 µm long); labium two-segmented, basal segment indicated by pair of setae on ventral surface; microtubular ducts absent from ventral surface of head	**8**
8	Anal ring with ≤ 6 setae; quinquelocular pores absent from venter; antennae six-segmented; venter extremely hirsute	***Lachnodiushirsutus* (Froggatt)**
–	Anal ring with > 10 setae; quinquelocular pores present on venter; antennae seven-segmented; venter not extremely hirsute	**9**
9	Venter with dense submarginal band of quinquelocular pores; marginal setae longer than anal ring setae	***Lachnodiusbrimblecombei* sp. n.**
–	Venter without dense submarginal band of quinquelocular pores; marginal setae shorter than anal ring setae	***Lachnodiuslectularius* Maskell**

### Lachnodius brimblecombei

Taxon classificationAnimaliaHemipteraEriococcidae

Beardsley, Gullan & Hardy sp. n.

http://zoobank.org/5A129F1F-3598-461D-9E50-50D4F8D3715D

Figs 1a , 3

#### Diagnosis.

Gall of adult female covers portion of dorsum; adult female with marginal fringe of close-set setae, each longer than anal ring setae; one size class of dorsal macrotubular ducts.

#### Description.

**Adult female** (n = 10). Body outline circular to oval; length 2.6–7.3 mm (4.9 mm for holotype), greatest width 2.3–4.9 mm (3.8 mm for holotype). Eyes 43–58 μm wide, on margin. Antennae seven-segmented; length 980–1380 μm; with 4–5 hair-like setae on segment I, 9–11 hair-like seta on segment II, 6–8 hair-like seta on segment III, 2–3 hair-like seta on segment IV, zero or one hair-like + one fleshy seta on segment V, two hair-like setae + one fleshy seta on segment VI and six hair-like setae + three fleshy setae on segment VII. Frontal lobes 250–300 µm long, 65–165 µm wide. Tentorial box 375–510 μm long, 200–280 μm wide, with anterior extension of the dorsal arms. Labium two-segmented, 160–210 μm long, 170–215 μm wide. Spiracles 190–240 μm long, 140–215 μm wide across atrium. Legs increasing in size caudad, fore leg: trochanter + femur 710–1060 μm, tibia 590–900 µm, tarsus 225–320 μm; mid leg: trochanter + femur 770–1150 μm, tibia 610–900 µm, tarsus 240–325 μm; hind leg: trochanter + femur 810–1260 μm, tibia 650–1040 µm, tarsus 260–400 μm; claw 63–90 μm; fore coxa with 6–8 setae, mid and hind coxae each with 5–7 setae, trochanter with 6–8 setae, femur with 15−31 setae, tibia with 19–38 setae, tarsus with 10–16 setae; tarsal digitules 83–100 μm long, claw digitules 50–73 μm long; translucent pores on all segments of hind leg. Anal ring 80–108 μm wide, with 12–16 setae; ring setae 70–115 μm long. Pair of elongate caudal setae absent.

*Dorsum*. Derm membranous. Dorsal setae 8−10 μm long, each parallel-sided, with acute apex, scattered over dorsum. Macrotubular ducts with rim of dermal orifice 5 µm in diameter, duct shaft 8–10 µm long, scattered over dorsum. Microtubular ducts ca. 5 μm long, with rim of dermal orifice ca. 2 μm wide, scattered over dorsum. Dorsum delimited by fringe of setae, each 70–118 µm long, ca. 200 setae in total on each side of body.

*Venter*. Larger than dorsum. Ventral setae 40–180 μm long; elongate setae medial of each coxa 150–225 μm long; longest setae on head 205–350 μm long. Macrotubular ducts similar to those on dorsum; in transverse band across each abdominal segment, scattered throughout submargin, medial of meso- and metacoxa. Quinquelocular pores 5 μm in diameter, found wherever setae occur, in transverse band across each segment, in a dense band along submargin, dense on posterior abdominal segments and around each spiracle.

**Second-instar female** (n = 9). Shape of slide-mounted specimen moderately elongate oval to broadly oval; length 1.9–3.0 mm, width 0.8–1.1 mm. Antennae six-segmented, short (230 µm total length), basally broad, becoming narrower toward apex, segment III ca. 60 µm wide. Legs short, broad, all segments present but tibiae and tarsi partially fused; tarsal claws incompletely developed. Anal ring ca. 30 µm wide, with ca. 10 setae, each of 25 µm maximum length. Dorsum with sparse, scattered setae, very small (mostly 4–5 µm long), acute or with blunt apices, and sparsely scattered, very small, tubular ducts ca. 2–3 µm in orifice diameter. Marginal fringe a moderately sparse series of 85–90 conical setae on each side of body, each seta ca. 35–50 µm long; antepenultimate seta of fringe on each side longer, ca. 80–90 µm long; fringe setae within a narrow marginal band of quinquelocular pores ca. 4–6 pores wide, extending around body; a small number of trilocular and quadrilocular pores scattered among quinquelocular pores. Venter with a few quinquelocular pores near each spiracle. Ventral setae flagellate, ranging from 10–65 μm long on thorax and abdomen, as long as 115 μm on head.

**Second-instar male** (n = 3). Shape of slide-mounted specimens moderately elongate-oval; length 1.2–1.5 mm. Antennae 7-segmented, ca. 280 µm long, slender, segment III 30–40 µm wide. Legs normal, slender, all segments present with tibiae 1.7–1.9 times length of tarsi; tarsal claws normally developed. Anal lobes narrowly separated by shallow anal cleft. Anal ring ca. 40 µm wide, with approximately 10 setae, 45 µm maximum length. Dorsum with derm finely spiculate. Dorsal setae sparse, scattered, short (ca. 4 µm long), acute, borne on papillae with narrow sclerotized rims. Dorsum with numerous (ca. 80) moderately large (ca. 8 µm rim and 6 µm orifice diameter) macrotubular ducts arranged in segmental rows. Marginal fringe of ca. 85 setae on each side, each seta moderately long (ca. 28–45 µm), conical with more or less filamentous apex; antepenultimate seta on each side much longer, to 105 µm; three most posterior setae on each side (including elongate antepenultimate) borne on a small, sclerotized anal lobe. Venter with a marginal line of quinquelocular pores just mesad of marginal fringe, around entire body, approximately as numerous as fringe setae; pores very sparsely scattered elsewhere on venter. Ventral tubular ducts absent. Ventral setae flagellate, ranging from 15–50 µm on thorax and abdomen, as long as ca. 125 µm on head.

#### Notes.

The slide-mounted adult female of *L.brimblecombei* is most similar to that of *L.lectularius*. Each has a marginal fringe of close-set setae, and the dorsum densely beset with macrotubular ducts of a single type, none of which have a seta touching the dermal orifice. In life the two are easy to distinguish. The adult female of *L.brimblecombei* induces a deep stem or bud gall with considerable swelling of the surrounding tissue that covers a portion of the female’s dorsum (Fig. 1a). If the gall occurs on the stem, it causes the stem to bend (Fig. 1a), often sharply. The adult female of *L.lectularius* also induces a gall on the stem or bud of the host, but the gall does not cover any portion of the female’s dorsum (Fig. 2a, b), and if on a stem does not make it crooked. Slide-mounted specimens of *L.brimblecombei* can be distinguished from those of *L.lectularius* by having (1) a dense marginal band of quinquelocular pores on the venter (absent in *L.lectularius*) and (2) the marginal setae longer than the anal ring setae (marginal setae shorter than anal ring setae in *L.lectularius*).

**Figure 3.**
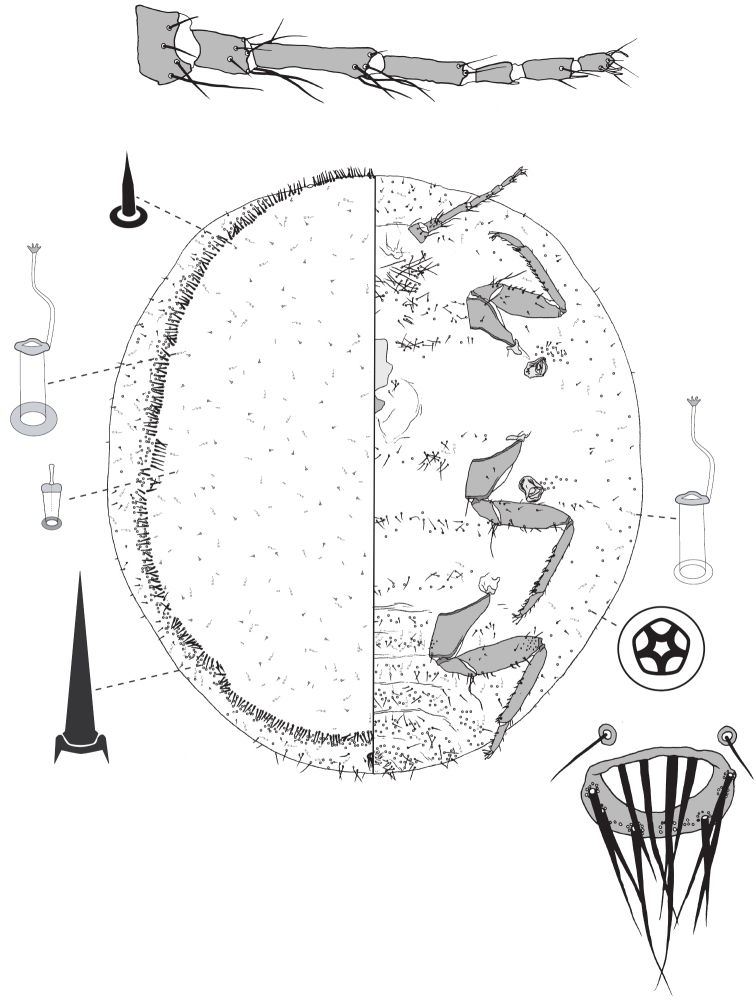
Adult female of *Lachnodiusbrimblecombei* sp. n.

The habitat of the Victorian specimens of *L.brimblecombei*, which develop in galls formed on flower buds, is different from that of the type specimens from Queensland (with galls as in Fig. 2a). But we found no significant morphological differences among specimens from the different states, except for slightly smaller fringe setae and possibly fewer quinquelocular pores in the Victorian specimens. In case further study reveals that the Victoria specimens are different, we have restricted the type series to specimens collected by AR Brimblecombe in Queensland and three adult females from New South Wales, one of which is a DNA voucher.

At Wilson’s Promontory in Victoria, galling caused by *L.brimblecombei* on *E.baxteri* reduces bud survival and flowering (Andersen 1989). Monitoring of tagged eucalypt shoots showed that, although less than 12% of buds were galled by *L.brimblecombei* (misidentified as *Opisthoscelis* sp.), the presence of galled buds often caused the abscission of nearby non-galled buds and galling on flowering stems often resulted in the loss of entire inflorescences, but these losses may be exacerbated by low water availability. On heavily galled shoots, the proportion of flowers producing mature fruit was correlated negatively with degree of galling, indicating that there was no compensatory increase in the success of the non-galled buds. Thus *L.brimblecombei* may decrease the fitness of its host, especially during periods of low rainfall.

A live adult female from Wild Cattle Creek State Forest in New South Wales was covered in white powdery wax and had a marginal fringe of white wax filaments ca. 0.2 mm long. Evidence of attack by parasitoid wasps was seen in several of the specimens studied. Two females from Redland Bay, Queensland, contained mandibles of parasitoid larvae, and the specimen from Mittagong, NSW also was parasitized.

We also examined one large (ca. 10 mm long) adult female that may be a developmentally abnormal specimen of *L.brimblecombei* or it might be a new species. It was collected from a stem pit on *E.fasciculosa* at Belair in South Australia (ANIC). It differs from typical adult females of *L.brimblecombei* in having reduced and distorted legs and antennae, many more dorsal fringe setae and in lacking the narrow marginal band of disc pores.

#### Etymology.

Pioneering Australian coccidologist AR Brimblecombe recognized this species and used the manuscript name ‘Lachnodius geniculatus’ to refer to it in his dissertation [citation of this name here is NOT intended to be for nomenclatural purposes; the name is not valid]. This species is named in Brimblecombe’s honor. The species epithet is a noun in the genitive singular.

#### Material examined.

***Holotype***: **Queensland**: adult female, on slide: ex pit gall in young twig of *Eucalyptusmicrantha*, Redland Bay, 2 Aug 1937, AR Brimblecombe, No. SC 147 (QM). ***Paratypes***: **Queensland**: 15 second-instar females (on three slides, including two on same slide as holotype), five second-instar males (on two slides with second-instar females): same data as holotype (QDPC, QM) [The slide of another adult female from the type series could not be located at QDPC.]; two adult females: *Eucalyptus* sp., Glasshouse Mts., Queensland, 20 Dec 1935, AR Brimblecombe (QDPC). **New South Wales**: one adult female: ex deep pocket gall in twig of *Eucalyptus* sp., Mittagong, 24 Nov 1899, WW Froggatt, #305 (ASCU); one adult female: ex stem depression, *Eucalyptus* sp., west side of Mt Jerrabomberra, 35.35S, 149.23E, 2 May 1993, LG Cook (ANIC); one adult female: ex stem gall, *Eucalyptus* sp. sapling, Wild Cattle Creek State Forest, above Platypus Flat, 30.18S, 152.70E, 11 Oct 1996, PJG, Lach4 of LGC (ANIC). ***Additional material***: **Victoria**: two adult females, four second-instar females: ex deep cavity galls in deformed flower buds, *E.baxteri*, Wilson’s Promontory, 8 Feb 1972, JWB (BPBM); one adult female, one second-instar female: ex deep galls in aborted flower buds, *E.baxteri*, Wilson’s Promontory, Squeaky Beach, 24 Feb 1972, A Yen (BPBM); two second-instar females, one second-instar male, three first-instar nymphs: ex bud galls, *E.baxteri*, Wilson’s Promontory, Tidal Overlook, 23 Sep 1982, AN Anderson (ANIC); eight adult females: same data as previous except 12 Nov 1982 (ANIC); one parasitized second-instar female (probably *L.brimblecombei*), ex pit in swollen stem, *E.baxteri*, Grampians, Wartook Valley, Emu Holiday Park, 37.06S, 142.33E, 10 Jan 2011, PJG (ANIC).

### Lachnodius eucalypti

Taxon classificationAnimaliaHemipteraEriococcidae

(Maskell, 1892)

Figs 1b, c , 4

Dactylopius eucalypti Maskell, 1892: 35; 1893: 233.
Lachnodius eucalypti : Maskell 1896: 400; Morrison and Morrison 1922: 44–48.

#### Diagnosis.

Loose marginal fringe with minute sagittate setae; microtubular ducts on venter of head; macrotubular ducts with distal attenuation.

#### Description.

**Adult female** (n = ca. 100). Body outline circular to oval; length 2.9–5.5 mm (3.5 mm for lectotype), greatest width 2.7–4.5 mm (3.0 mm for lectotype). Eyes 40–56 μm wide, on margin. Antennae seven-segmented; length 450–740 μm; with 2–3 hair-like setae on segment I, 4–10 hair-like seta on segment II, 2–6 hair-like seta on segment III, 4–7 hair-like seta on segment IV, 2–4 hair-like + one fleshy seta on segment V, 3–5 hair-like setae + one fleshy seta on segment VI and six hair-like setae + three fleshy setae on segment VII. Frontal lobes 210–300 µm long, 85–200 µm wide. Tentorial box 205–360 μm long, 175–265 μm wide, with anterior extension of the dorsal arms. Labium 90–125 μm long, 110–135 μm wide, one-segmented, proximal segment setae absent. Spiracles 110–175 μm long, 60–115 μm wide across atrium. Legs: trochanter + femur 400–660 μm, tibia 260–450 µm, tarsus 110–150 μm; claw 38–53 μm; fore coxa with six setae, mid and hind coxae each with five setae, trochanter with 4–8 setae, femur with 6–18 setae, tibia with 12–19 setae, tarsus with 4–9 setae; tarsal digitules 63–90 μm long, claw digitules 48–65 μm long; translucent pores on all segments of hind leg. Anal ring 78–115 μm wide, with 18–30 setae; ring setae 60–155 μm long. Pair of elongate caudal setae absent.

*Dorsum*. Derm membranous. Dorsal setae each parallel-sided, with acute apex, 5–7 μm long, scattered over dorsum. Macrotubular ducts with rim of dermal orifice 5 µm in diameter, duct shaft 13–20 µm long, distal portion (subtending vestibule) constricted, scattered over dorsum. Microtubular ducts ca. 5 μm long, with rim of dermal orifice ca. 2 μm wide, scattered over dorsum. Dorsum delimited by fringe of alternating minute sagittate setae, each 6–18 µm long, and slightly larger setae, 10–20 µm long, ca. 150 setae in total on each side of body.

*Venter*. Ventral setae 10–75 μm long; elongate setae medial of each coxa 40–115 μm long; longest setae on head 120–150 μm long. Macrotubular ducts similar to those on dorsum; in transverse band across each abdominal segment, scattered throughout submargin. Quinquelocular pores 5 μm in diameter, clustered around vulva and each spiracle, present wherever setae found. Microtubular ducts on head.

**First-instar nymph** (n = 14 from Bundoora, Victoria). This instar was redescribed and figured well by Morrison and Morrison (1922, figure 14) and only some additional information is provided here. Newly hatched individuals ca. 380–400 µm long; feeding first-instar nymphs removed from leaf galls 550–600 µm long, broadly oval in outline, with venter expanded, balloon-like, to fill gall cavity, dorsum flat. Slide-mounted specimens with medial to submedial dorsal derm bearing small sclerotic spots, mostly 1–2 µm in greatest dimension; marginal setae mostly falcate (incorrectly described as ‘flabellate’ by Morrison & Morrison) except posterior three pairs lanceolate but often with apex jagged or notched, each marginal seta 15–30 µm long. Pair of elongate caudal setae ca. 65 µm long.

#### Notes.

The adult female of *L.eucalypti* could be confused most easily with that of *L.froggatti* sp. n. Each induces pit galls on leaves and may be covered by waxy secretions. The adult female of *L.eucalypti* differs from that of *L.froggatti* by having (1) a marginal fringe of alternating sagittate and conical setae (marginal setae of *L.froggatti* hair-like to capitate); (2) eyes on margin (eyes on venter of *L.froggatti*); (3) no dorsal macrotubular ducts with setae touching rim of dermal orifice (dorsum of *L.froggatti* having some macrotubular ducts with a seta touching dermal orifice); and (4) microtubular ducts on ventral surface of head (absent in *L.froggatti*). Also, in life the secretions covering an adult female of *L.froggatti* are woolly, in contrast to the clumpy, powdery secretions that cover an adult female of *L.eucalypti*. Populations of *L.eucalypti* are known from all eight Australian states and territories. Specimens of *L.eucalypti* have been collected most commonly from *E.camaldulensis*, which is the most widely distributed species of *Eucalyptus* in Australia (Brooker 2002), but they also have been taken from a number of additional species of *Eucalyptus* in three sections (*Adnataria*, *Exsertaria*, and *Maidenaria*) of the subgenus Symphyomyrtus. Two populations of adult females probably both from *E.camaldulensis* (Windjana Gorge in northern Western Australia and near Alice Springs in the Northern Territory) have the sagittate setae of the marginal fringe of more uniform length and larger (15–20 µm long) compared with populations from the eastern and southern states in which the sagittate setae vary in size from 6–18 (mostly < 15) µm long on individual specimens. Due to this difference, we have excluded the females collected in the Northern Territory and Western Australia from the description above. Freshly collected specimens suitable for DNA sequencing might allow a decision on the species status of this morphological variation.

Life history data for *L.eucalypti* were obtained by JWB from a population that infested mature trees of *E.camaldulensis* on the campus of La Trobe University, Bundoora, Victoria, during the spring, summer, and fall of 1971–72. Beginning on 29 September 1971, adult females of *L.eucalypti* were collected while ovipositing on the bark of trunks and major branches of host trees. Oviposition was intermittent between then and mid-February 1972. Individual females appeared to complete oviposition within a short period of two or three days. The eggs were pink and laid in a single layer that formed a long, flat ribbon, 4–6 eggs wide, the top and sides of which were enclosed by a waxy secretion (Fig. 1c). Individual ovisacs were sometimes more than 5 cm long, straight or curved, and contained on the order of several hundred eggs (although no counts were made). A shrivelled, moribund female was often found at the end of an ovisac.

In the laboratory, eggs hatched 7–10 days after deposition. On host trees, the newly-eclosed first-instar nymphs migrated from the oviposition sites to the foliage, where they settled on the upper surfaces of young leaves. Feeding by each nymph resulted in a shallow pit gall on the leaf surface, which enclosed the nymph and grew along with it. The dorsal surface of settled first-instar and second-instar nymphs was nearly flat, smooth, and shiny, without evident waxy secretions. The ventral part of the nymph’s body filled the cavity of the pit gall, while the dorsal margin overlapped and sealed the edge of the gall cavity.

In second-instar females the legs are poorly developed and apparently non-functional. Male nymphs, which can be distinguished from females in the second instar by the presence of fully developed legs, developed in leaf galls similar to those of females. Second-instar males eventually abandoned their galls and migrated to the bark of trunks and branches of host trees where they formed ovoid cocoons in protected situations. In the laboratory, males formed cocoons under paper lining the bottom of the petri dishes in which they were held. Cocoons were formed of whitish filaments, which issued from the dorsal tubular ducts.

Females remained in their galls after molting to the third (adult) instar, and continued to feed for an undetermined period, until fully developed. They then abandoned their galls and migrated to the bark to oviposit. When and where mating took place was not determined. At La Trobe University, the population of *L.eucalypti* did not appear to reproduce synchronously. Although ovipositing females were observed only during the spring and summer months (September to February), individuals of all stages were found on the trees during late January.

Maskell (1892) described this species based on adult females, pupal and adult males, and first-instar nymphs, collected from a tree referred to as *E.amygdalina*. The following year, Maskell (1893) indicated that his type material of *Dactylopiuseucalypti* was from South Australia, and that the specimens were collected under bark. It appears that Maskell received the type material from the South Australian collections accumulated by Frazer S Crawford of Adelaide, an economic entomologist with an interest in Coccoidea. However, the identification of the host tree as *E.amygdalina* is problematic if the insects came from South Australia, because this eucalypt is endemic to Tasmania. Specimens of a second collection, which Maskell received from WW Froggatt in Sydney, were in pit galls in the leaves of *E.robusta*. This difference in the site of collection on the host trees apparently gave Maskell the impression that the species developed both in leaf galls and under bark, and presumably he was unaware that adult females migrate from leaf galls to bark prior to oviposition.

The Maskell collection in the NZAC contains six slides of *L.eucalypti*, four of which we consider to be type material. The four slides with type specimens are labeled “*Dactylopius eucalypti*” with the word “*Dactylopius*” crossed out and “*Lachnodius*” written above it. These labels also have the locality as “Australia” and the date as “1886.” The slides bear (1) an adult female, (2) an adult male, (3) three first-instar nymphs, and (4) adult male parts (part of the thorax, two antennae, and two legs). The other two Maskell slides of this species in the NZAC contain (1) an adult female and (2) eight first-instar nymphs and bear later collection data (1893 and 1894) and therefore could not have been part of the material on which Maskell based his description. Beardsley had intended to designate the 1886 slide bearing the adult female as the lectotype of *Dactylopiuseucalypti* Maskell, and labelled it as such in 1972 but this action was not published until now.

Note that there are also two slides of first-instar nymphs from the Maskell collection in the USNM, apparently from the type lot. Morrison and Morrison (1922: 44, 46) referred to one collection as “… a very small amount of material in position on the host, under Maskell No. 206” and listed the other slide as “Cotype. – Cat. No. 24762, U.S.N.M.”. We examined both slides and list them below as paralectotypes.

#### Material examined.

***Lectotype*** (here designated): adult female: on slide labelled: “*Lachnodius* / *Dactylopius* / *eucalypti* / adult female / Australia / 1886 W.M.M.” (ANIC). ***Paralectotypes***: one adult male (one slide), antenna and feet of adult male (one slide), three first-instar nymphs (one slide, labelled “Larvae”), same data as lectotype (NZAC); 12 first-instar nymphs: on slide labelled: “*Lachnodius* / *eucalypti* (Mask.) / Australia / Mask. Coll. N. 82 / Type” and envelope also with “Cotype Cat. No. 24962 / U. S. National Museum” (USNM); two first-instar nymphs: on slide labelled: “*Lachnodius* / *eucalypti* (Mask.) / Australia / Mask. Coll. 206 (USNM). ***Additional material***: **Unspecified locality in Australia**: one adult female: same label data as lectotype except “1893” and “not type, described 1892 / L. L. Deitz 1978” [JWB erroneously added a paralectotype label] (NZAC); eight first-instar nymphs: same label data as lectotype except “1894” and “not type, described 1892” (NZAC); one adult female: ex *Eucalyptuscamaldulensis*, quarantine intercept in Cambridge, UK, 1 Nov 1993, Newman, 93-1216 (ANIC). **Australian Capital Territory**: three adult females, 14 first-instar nymphs on three slides: ex trunk, *E.mannifera*, Charnwood (suburb), Canberra, 18 Nov 2015, PJG (ANIC); one adult female (parasitized), 65 first-instar nymphs on eight slides: ex pit gall on leaf, *E.bridgesiana*, Tidbinbilla Nature Reserve, 35.48S, 148.89E, 1 Mar 1992, PJG (ANIC). **New South Wales**: 15 adult females: ex pits in leaves, *E.blakelyi*, 6.5 km SE of Forbes, 28 Nov 1984, PJG (ANIC); one adult female (parasitized): under bark, *E.viminalis*, Bago State Forest, 10 km ESE of Batlow, 14 Jan 1979, PJG (ANIC); three adult females: ex foliage, *Eucalyptus* sp. (ironbark), nr Coonabarabran, Warrumbungle Nat. Park, Camp Pincham, 22 Nov 1985, CAM Reid (ANIC); one adult female: ex pit gall on leaf, *E.saligna*, S. Brooman, “Strathclyde” (property), bank of Clyde River, 35.52S, 150.22E, 10 Jan 1996, PJG (ANIC); one adult female: Dubbo, no date, Froggatt #1049 [JWB must have misread the Froggatt number as this collection matches #1079 for *L.eucalypti*: “WWF 20.11.1921 / Dubbo / *Eucalyptus*”] (ASCU); partial specimens of adult and second-instar females: ex pit galls, E. *botryoides* leaves, Kurnell, 26 Aug 1915, WW Froggatt, #621 (ASCU); one adult female: ex leaf pit, ?*E.tereticornis* or *E.seeana* , South West Rocks, 30.90S, 153.02E, 30 Dec 2009, LG Cook, LGC01374 (ANIC); one adult female: in leaf pit gall, *E.tereticornis*, Wagga Wagga, 6 Nov 1899, WW Froggatt, # 297 (ASCU). **Northern Territory**: three adult females: *Eucalyptus* sp., N of Alice Springs, near Todd River, 19 Nov 1978, M Kotzman (ANIC). **Queensland**: six adult females: ex *E.propinqua*, Imbil, Oct 1936, AR Brimblecombe, No. D2264-6 (QDPC); three adult females, three second-instar males, five second-instar females: ex foliage, *Eucalyptus* sp., N side of Tamborine Mt, nr Sandy Creek, 26 Sep 1989, PJG (ANIC). **South Australia**: 15 adult females, two adult males, many first-instar nymphs, 14 second-instar males, six pupal males: *E.camaldulensis*, Adelaide, Glen Osmond, Waite Agric. Res. Instit., Dec 1952, HM Brookes, HMB Specimen Index No. 77/54 (ANIC); five adult females, one second-instar female: ex pits in leaves, *E.camaldulensis*, Glen Osmond, 27 Jul 1965, HMB, HMB Specimen Index No. 21/65 (ANIC); six adult females: on *E.camaldulensis*, 1 mile [1.6 km] N of Greenock, 19 Dec 1960, HM Brookes, HMB Specimen Index No. 109/60 (ANIC); 28 adult females: on bark of *E. ?camaldulensis*, Hazelwood Park, 14 Nov 1966, RS Bungey, HMB Specimen Index No. 46/66 (ANIC); four adult females: ex pits in leaves of *E.camaldulensis*, Mt Crawford Forest Reserve, Jan 1982, HM Brookes, HMB Specimen Index No. 1/82 (ANIC); two adult females, four second-instar females: *E.microtheca*, nr Murnpeowie (homestead), 16 Aug 1968, FD Morgan (ANIC); one adult female: under bark, *E.camaldulensis*, nr Mt Barker township, 15 Dec 1985, CAM Reid (ANIC); one adult female, six adult males: under bark, *E.camaldulensis*, Sampson Flat, 7 Sep 1965, DC Purdie (ANIC). **Tasmania**: two adult females: *E.globulus*, Hobart, Sandy Bay, 21 Aug 1965, HMB (ANIC). **Victoria**: one adult female, six second-instar females, one second-instar male, six first-instar nymphs: ex pit galls on young leaves, *E.camaldulensis*, Bundoora, La Trobe University, 24 Jan 1972, JWB (BPBM); one adult female (parasitized mummy): ex pit gall on twig, same data as previous except 5 Jan 1971 JWB (BPBM); two adult females, eight first-instar nymphs: on bark, same data as previous except 29 Sep 1971, V-87 (BPBM except one slide with three nymphs in ANIC); 16 adult females: same data as previous except 10 Dec 1971, V-243 (BPBM); one adult female, two adult males: same data as previous except Oct 1971 (BPBM); three adult females, four second-instar males: on bark, *E.goniocalyx*, Melbourne, Lower Plenty, 11 Sep 1971 or 23 Sep 1971, JWB (BPBM); one adult female: on stem, *E.camaldulensis*, W of Benalla, 36.48S, 145.95E, 26 Nov 2006, PJG, NH150 (ANIC); five adult females, two second-instar females: on *E.nitens*, Errinundra Plateau, Orbost Forestry District, 21 Oct 1974, FG Neumann and GC Marks, HMB Specimen Index No. 14/74 (ANIC). **Western Australia**: 21 adult females, one second-instar female with pharate adult: ex gall, *E.camaldulensis*, Windjana Gorge Nat. Park, bank of Lennard River, 17.42S, 124.95E, 29 Apr 1992, PJG (ANIC except eight slides in WAM).

**Figure 4.**
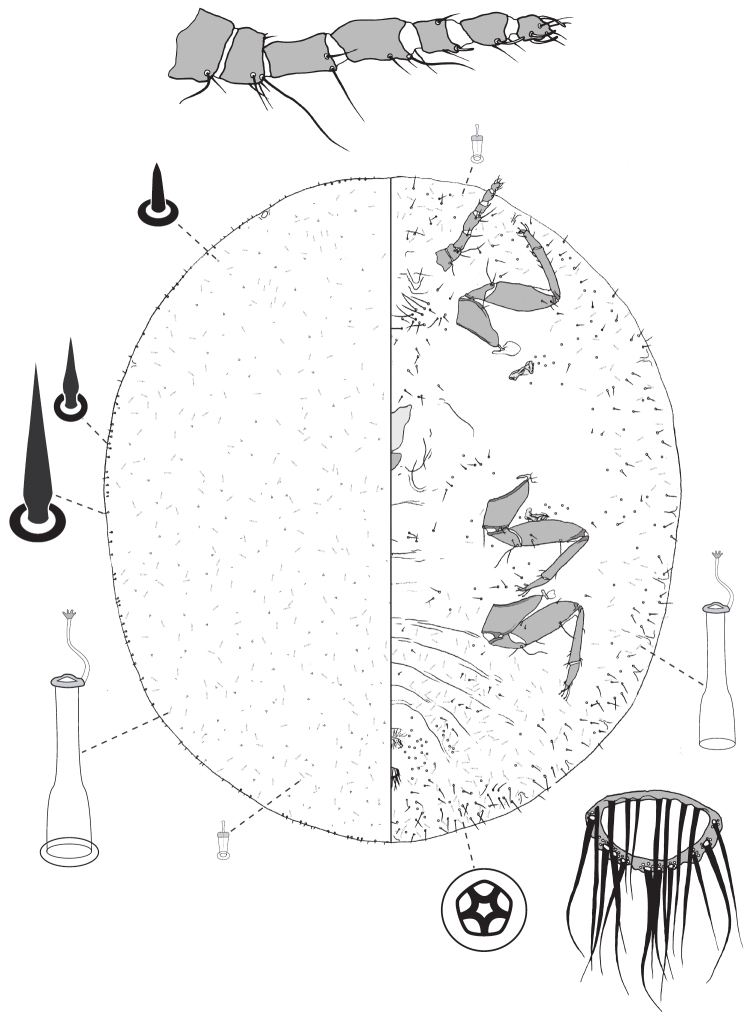
Adult female of *Lachnodiuseucalypti* (Maskell).

### Lachnodius froggatti

Taxon classificationAnimaliaHemipteraEriococcidae

Beardsley, Gullan & Hardy sp. n.

http://zoobank.org/2869D8E4-DDEE-4563-99C0-09F8A867FDFD

Figs 1 d, e , 5

#### Diagnosis.

Eyes on venter; dorsal derm membranous; two size classes of dorsal marotubular ducts, some larger ducts with seta touching rim.

#### Description.

**Adult female** (n = 30). Body outline circular to oval; length 2.3–8.9 mm (5.5 mm for holotype), greatest width 1.8–5.8 mm (4.3 mm for holotype). Eyes 47–75 μm wide, on venter between margin and scape. Antennae seven-segmented; length 760–1580 μm; with 6–9 hair-like setae on segment I, 8–21 hair-like seta on segment II, 20–28 hair-like seta on segment III, 10–18 hair-like seta on segment IV, 3–9 hair-like + one fleshy seta on segment V, 4–7 hair-like setae + one fleshy seta on segment VI and six hair-like setae + three fleshy setae on segment VII. Frontal lobes 150–340 µm long, 75–190 µm wide. Tentorial box 270–480 μm long, 200–330 μm wide, with anterior extension of the dorsal arms. Labium 110–155 μm long, 135–230 μm wide. Spiracles 140–305 μm long, 75–190 μm wide across atrium. Legs: trochanter + femur 545–1080 μm, tibia 420−940 µm, tarsus 150–270 μm; claw 43–70 μm; fore coxa with six setae, mid and hind coxae each with five setae, trochanter with 5–9 setae, femur with 20–40 setae, tibia with 18–51 setae, tarsus with 7–15 setae; tarsal digitules 63–98 μm long, claw digitules 45–68 μm long; translucent pores on all segments of hind leg. Anal ring 83–148 μm wide, with 18–29 setae; ring setae 100–225 μm long. Pair of elongate caudal setae absent.

*Dorsum*. Derm membranous. Dorsal setae 5–10 μm long, each parallel-side, with acute apex, scattered over dorsum. Macrotubular ducts of two size classes: (1) large ducts with rim of dermal orifice 8–10 µm in diameter, sometimes with seta touching rim, duct shaft 20–30 µm long, scattered over dorsum; (2) smaller ducts, rim of dermal orifice 5–6 µm in diameter, duct shaft 10–20 µm long, scattered over dorsum. Microtubular ducts ca. 5 μm long, with rim of dermal orifice ca. 2 μm wide, scattered over dorsum. Dorsum delimited by fringe of setae, each 18–53 µm long, ca. 200 setae in total on each side of body.

*Venter*. Ventral setae 18–183 μm long; elongate setae medial of each coxa 120−340 μm long; longest setae on head 185−365 μm long. Macrotubular ducts similar to those on dorsum; found wherever setae occur, in transverse band across each segment, scattered throughout submargin. Quinquelocular pores 5 μm in diameter, sparse, distributed as for macrotubular ducts, with cluster near each spiracle and caudad of vulva.

**Second-instar female** (n = 5). Broadly oval to nearly circular in outline; length 1.7–3.2 mm. Eyes ca. about one eye diameter removed from fringe line on venter. Antenna six-segmented, ca. 190 µm long, strongly tapered base to apex, segments except apical broader than long. Legs short and broad, all segments differentiated, claws vestigial. Anal ring ca. 35 µm wide, with ca. eight setae to ca. 36 µm long. Dorsum with small setae (4–8 mu long), sparse, spiniform. Dorsal macrotubular ducts, ca. 5 µm orifice diameter, 8 µm rim diameter, ca. 18–20 µm long, some with a satellite seta, sparsely scattered in submarginal band around periphery of body; minute tubular ducts (ca. 2 µm orifice diameter) interspersed among larger ducts. Marginal fringe a moderately sparse series of moderately slender conical setae, 18–28 µm long, with apices blunt or very slightly expanded; ca. 90 setae on each side. Antepenultimate setae slightly longer (30–40 µm long). Venter with very sparse setae, mostly 20–30 µm long, 40–50 µm between legs, to 75 µm on head. Ventral macrotubular ducts absent. Ventral quinquelocular pores sparsely scattered in submarginal peripheral band, plus slight concentrations near spiracles.

#### Notes.

The adult female of *L.froggatti* is most similar to that of *L.eucalypti*. See notes for *L.eucalypti* for a comparison. Populations of *L.froggatti* have been sampled from New South Wales, Victoria, and South Australia. It is known to feed on hosts in the subgenera Eucalyptus (sectionEucalyptus) and *Symphyomytrus* (sections *Adnataria* and *Maidenaria*). The live adult female is white to pale cream or yellow in life, and mature females produce copious dorsal glassy wax filaments and white powdery wax (Fig. 1d, e). The females have been found only on the leaves and the pit below the female’s body may be up to 1.5 mm deep (Fig. 1e). The leaf area around the feeding insect is often depressed and discolored or necrotic, and the opposite surface of the leaf has a bulge; on very young foliage, the female causes leaf curling.

Froggatt’s first accession notebook (Gullan 1984a) has an entry for the specimen that we have designated as holotype, as follows: “(303) *Dactylopius eucalypti* ?Large funnel leaf Penrith (No 1) (Berlese No 233)”. The words “?Large funnel leaf” are written in different handwriting and inserted in the original entry. The mention of a Berlese number refers to part of this collection being sent to Berlese (presumably the Italian coccidologist Antonio Berlese) as a previous entry says “(Sent to Berlese No 230)”. It seems that Froggatt confused *L.froggatti* with *L.eucalypti*, as shown by his identification of our holotype of *L.froggatti* (discussed above) as *L.eucalypti*, and also the following record. Two paratype females listed below have a Froggatt number of 27, which Froggatt’s first accession notebook records as from Wallsend, which is one of the localities listed by Froggatt (1917, 1921) for *L.eucalypti*. We have restricted the type series of *L.froggatti* to specimens from New South Wales. All specimens in the Froggatt collection are from this state.

#### Etymology.

This species is named in honor of the collector of the type material, the late WW Froggatt, an Australian entomologist employed by the New South Wales Department of Agriculture during the early decades of the 20^th^ century. Froggatt was the first to seriously attempt a systematic treatment of the scale insect fauna of Australia. The species epithet is a noun in the genitive singular.

#### Material examined.

***Holotype***: **New South Wales**: adult female, on slide: ex open top pit gall on leaf, *Eucalyptus* sp., Penrith, 24 Nov 1899, W. W. Froggatt collection # 303 (ASCU); this specimen was removed from a dry gall and slide-mounted by JWB in April 1972. ***Paratypes***: **New South Wales**: two adult females: ex leaf pit galls, *Eucalyptus* sp., WW Froggatt number 27 [from Wallsend, see note above], ASCTHE101355, ASCTHE101356 (ASCU); one adult female, three second-instar females: ex pits on leaves, *Eucalyptus* sp., 10 km S of Coonabarabran, roadside verge, 29 Nov 1984, PJG (ANIC); one second-instar female, ex pit in leaf of *E.baueriana*, ca. 6 km WSW of Narooma, Wagonga Scenic Drive, 36.24S, 150.97E, 31 Dec 2008, PJG (ANIC); two adult females, one second-instar female with pharate adult: ex pits in leaves, *E. ?melliodora*, Oallen, 1760 Oallen Ford Road, Windellama, 35.13S, 150.02E, 10 Jan 2018, PJG (ANIC). ***Additional material*: South Australia**: ten adult females, eleven first-instar nymphs: ex pits on leaves, *E.viminalis*, Adelaide, Glen Osmond, Waite Agric. Res. Institute, 3 Oct 1967, NC Stewart, HMB Specimen Index No. 31/67 (ANIC); two adult females, one adult male: ex pits on leaves, *E.fasciculosa*, Belair, National Park, 1 Nov 1963, TCR. White, HMB Specimen Index No. 48/63 (ANIC); three adult females: ex pits in leaves, *Eucalyptus* sp., Mannum, Jan 1971, P Allen (ANIC); one adult female: ex pit on leaf, *E.obliqua*, Netherby, 4 Jan 1964, PG Martin, 2/64 (ANIC); two adult females: ex pits on leaves, *E.obliqua*, Netherby, 28 Nov 1963, SW Brown, HMB Specimen Index No. 70/63 (ANIC). **Victoria**: one adult female: ex pit in leaf, *E. ?microcarpa*, 10 km S of Nagambie, on road to Avenel, 36.38S, 145.17E, 7 Feb 2004, PJG, LGC00107 (ANIC); one adult female: ex pit in leaf, *E.microcarpa*, 10 km S of Nagambie, on road to Avenel, 36.38S, 145.17E, 30 Jan 2005, PJG, NH118 (ANIC); four adult females: ex pits on leaves, *E.melliodora*, 9 km N of Nagambie, Weir Road, 500 m W of Hwy M39, 36.70S, 145.17E, 2 Jan 2003, PJG, NH156 (ANIC); ten first-instar nymphs (no associated adult females but of same morphology as nymphs from Adelaide listed above): ex pits on leaves, *Eucalyptus* sp. (mallee), Hattah Lakes Nat. Park, 30 Apr 1972, JWB (BPBM except one slide with four nymphs in ANIC); three adult females: ex leaf pits in leaf curls, *E.largiflorens*, Mildura, River Road, Apex Park, near Murray River, 34.16S, 142.16E, 4 Feb 2005, NBH and PJG, NH39, NH116, NH149 (ANIC).

**Figure 5.**
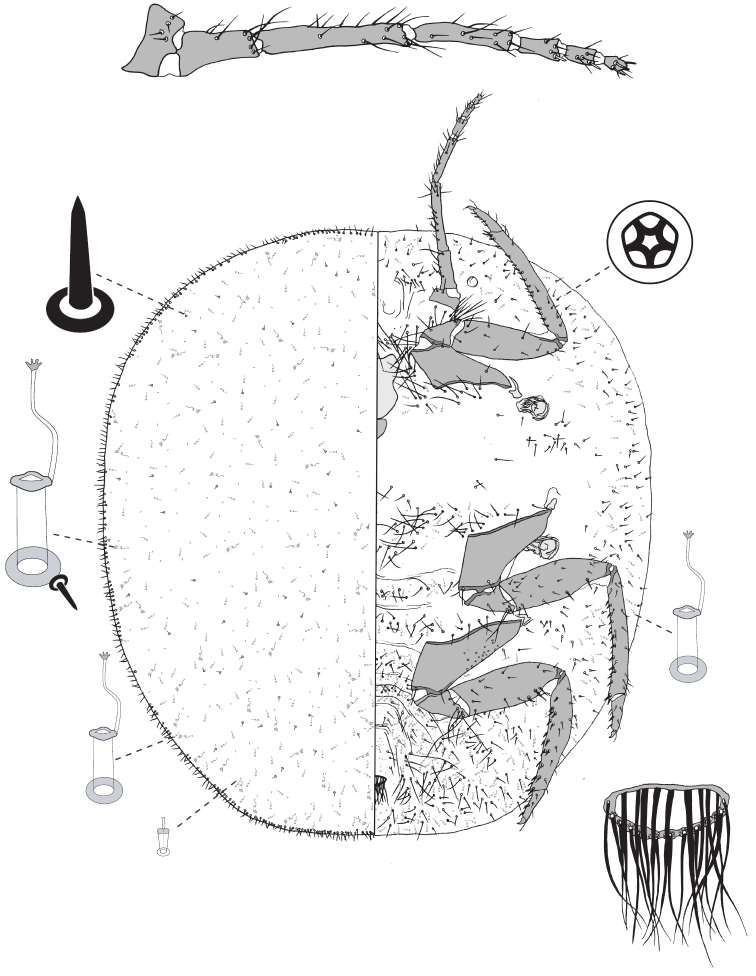
Adult female of *Lachnodiusfroggatti* sp. n.

### Lachnodius hirsutus

Taxon classificationAnimaliaHemipteraEriococcidae

(Froggatt, 1921)

Figs 1f , 6

Pseudopsylla hirsutus Froggatt, 1921: 6.
Lachnodius hirsutus Beardsley, 1982: 31–35.

#### Diagnosis.

Gall of adult female covers portion of dorsum; venter hirsute; anal ring with few setae and pores; microtubular ducts absent.

#### Description.

**Adult female** (n = 6). Body outline circular to oval; length 3.2–6.8 mm (3.2 mm for lectotype), greatest width 3.1–6.1 mm (3.1 mm for lectotype). Eyes 75–105 μm wide, on margin. Antennae six-segmented; length 850–1320 μm; with 3–4 hair-like setae on segment I, 8–18 hair-like seta on segment II, 8–11 hair-like seta on segment III, 9–15 hair-like seta on segment IV, 5–6 hair-like + one fleshy seta on segment V, and six hair-like setae + three fleshy seta on segment VI. Frontal lobes 275–750 µm long, 200–400 µm wide. Tentorial box 600–950 μm long, 200–450 μm wide, with anterior extension of the dorsal arms. Labium 200–270 μm long, 210–290 μm wide. Spiracles 250–360 μm long, 190–300 μm wide across atrium. Legs robust, increasing in size caudad, fore leg: trochanter + femur 940–1440 μm, tibia 810–1260 µm, tarsus 350–500 μm; mid leg: trochanter + femur 1020–1460 μm, tibia 890–1280 µm, tarsus 370–520 μm; hind leg: trochanter + femur 1280–1620 μm, tibia 1100–1380 µm, tarsus 500–560 μm; claw 110–160 μm; coxa with 20–44 setae, trochanter with 20–31 setae, femur with 30–70 setae, tibia with 37–75 setae, tarsus with 20–30 setae; tarsal digitules 80–95 μm long, claw digitules 68–85 μm long; translucent pores on all segments of hind leg. Anal ring 130–140 μm wide, ring thickening caudad, with 5–7 setae; ring setae 50–80 μm long. Pair of elongate caudal setae absent.

*Dorsum*. Derm membranous, nodulose. Dorsal setae 13–25 μm long, each tapering evenly from base to apex, scattered over dorsum. Macrotubular ducts with rim of dermal orifice 5–6 µm in diameter, duct shaft 15–18 µm long, distal (near vestibule) end constricted, ducts scattered over dorsum. Microtubular ducts absent. Dorsum delimited by dense fringe of elongate setae, each 200–455 µm long, ca. 250 setae in total on each side of body.

*Venter*. Ventral setae 75–210 μm long, distributed densely; elongate setae medial of each coxa 170–305 μm long; longest setae on head 260–360 μm long. Macrotubular ducts similar to those on dorsum, found wherever setae occur, in transverse band across each segment, and along submargin. Quinquelocular pores absent.

#### Notes.

The adult female of *L.hirsutus* can be distinguished from all other species by the combination of 6-segmented antennae, extremely long marginal setae (350–450 µm long), and the scarcity of quinquelocular pores, which occur only near the spiracular openings. The anal ring of *L.hirsutus* is also unique among *Lachnodius* species; it has six or fewer ring setae present, with only a few minute pores near the base of each seta.

In his redescription of this species, Beardsley (1982) omitted the length of the fourth segment from the antennal formula. The correct segment lengths (µm), from the base to the apex, are: 150, 120, 400, 200, 130, and 50. Froggatt (1921: 6) stated “The female coccids produce solid woody galls on the branchlets of eucalypts with an irregular opening on the upper surface (Fig. 1f). At female maturity, the gall of *L.hirsutus* probably splits open at the apex to reveal the female, because enclosed globular twig galls of nymphs have been collected in association with galls resembling those of *L.hirsutus* (Gullan et al. 2005). It is not clear whether the host genus of Froggatt’s type material was *Corymbia* or *Eucalyptus*, since the original description simply says “an undetermined species of eucalyptus [sic]”. The bloodwood eucalypts were not recognized as a genus (*Corymbia*) separate from *Eucalyptus* until more recently (Hill and Johnson 1995).

#### Material examined.

***Lectotype*** [designated by Beardsley (1982)]: **Northern Territory**: adult female, on slide: ex open top twig gall, *Eucalyptus* sp., Port Darwin, G. F. Hill, Froggatt # 629, ASCTHE101343 (ASCU); this specimen was remounted from an original Froggatt slide by JWB in April 1972. ***Paralectotype***: **Northern Territory**: one adult female: same data as lectotype, ASCTHE101342 (ASCU). ***Additional material***: **Northern Territory**: three adult females: ex galls on stems, *Corymbianesophila*, Gunn Point, 9 July 1987, LR Miller (ANIC); Queensland: one adult female: ex gall on stem, *E.tetradonta*, Iron Range Nat. Park, 4.2 km E of Cooks Hut campground, on road to Portland Roads, 79 m, 12.71S, 143.32E, 21 Sep 2006, LG Cook, LGC00642 (body with 2 intact legs), NH122, NH151, NH159 (NH numbers are for individual DNA-extracted legs) (ANIC).

**Figure 6.**
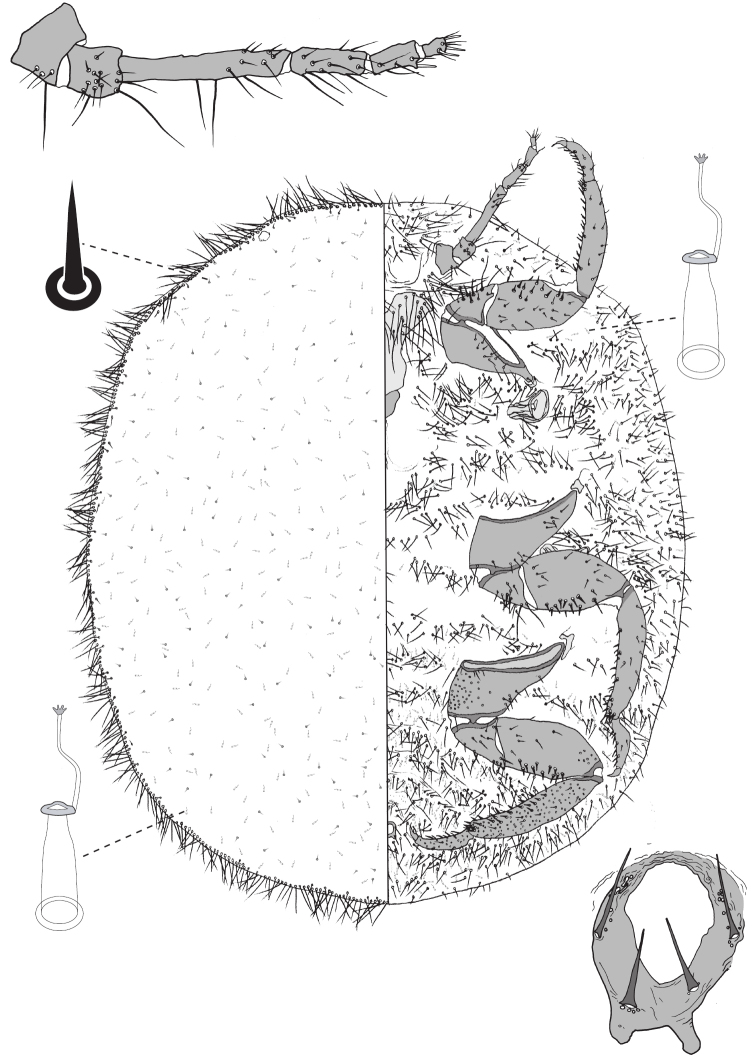
Adult female of *Lachnodiushirsutus* (Froggatt).

### Lachnodius lectularius

Taxon classificationAnimaliaHemipteraEriococcidae

Maskell, 1896

Figs 2a, b , 7

Lachnodius lectularius Maskell, 1896: 400–402.

#### Diagnosis.

Gall of adult female does not cover any of dorsum; adult female with marginal fringe of close-set setae; one size class of dorsal macrotubular ducts.

#### Description.

**Adult female** (n > 10). Body outline circular to oval; length 2.1–9.3 mm (4.0 mm for lectotype), greatest width 1.9–7.4 mm (3.0 mm for lectotype). Eyes 25–50 μm wide, on margin. Antennae seven-segmented; length 620−1440 μm; with 3–6 hair-like setae on segment I, 5–13 hair-like seta on segment II, 3–5 hair-like seta on segment III, 2–6 hair-like seta on segment IV, 2–3 hair-like + one fleshy seta on segment V, 2–4 hair-like setae + one fleshy seta on segment VI and six hair-like setae + three fleshy setae on segment VII. Frontal lobes 155–440 µm long, 88–175 µm wide. Tentorial box 330−500 μm long, 180–270 μm wide, with anterior extension of the dorsal arms. Labium 140–250 μm long, 120–190 μm wide. Spiracles 140–290 μm long, 100–285 μm wide across atrium. Legs: trochanter + femur 500–1340 μm, tibia 370–1150, tarsus 150−300 μm; claw 53–120 μm; coxa with 5–10 setae, trochanter with 6–14 setae, femur with 13–35 setae, tibia with 19–41 setae, tarsus with 11–21 setae; tarsal digitules 70–125 μm long, claw digitules 50–70 μm long; translucent pores on all segments of hind leg. Anal ring 73–185 μm wide, with 15–24 setae; ring setae 43–210 μm long. Pair of elongate caudal setae absent.

*Dorsum*. Derm membranous. Dorsal setae 5–8 μm long, each with constriction near base and apex acute, scattered over dorsum. Macrotubular ducts with rim of dermal orifice 5 µm in diameter, duct shaft 10–14 µm long, scattered over dorsum. Microtubular ducts ca. 5 μm long, with rim of dermal orifice ca. 2 μm wide, scattered over dorsum. Dorsum delimited by fringe of setae, each 38–90 µm long, ca. 300 setae in total on each side of body.

*Venter*. Ventral setae 15–210 μm long; elongate setae medial of each coxa 60–190 μm long; longest setae on head 165–300 μm long. Macrotubular ducts similar to those on dorsum; found wherever setae occur, in transverse band across each segment, scattered throughout submargin. Quinquelocular pores 5 μm in diameter, distributed as macrotubular ducts, with cluster near each spiracle, dense on median of posterior abdominal segments, near vulva.

#### Notes.

Adult females feed in a pit in a swollen stem or bud of the host eucalypt (Fig. 2a, b). The body color is variable; it is green with a red longitudinal stripe on the dorsum of younger females and fully orange or red to brown in older females. In life, females can lift up their abdomen and expose their venter. Each seta forming the marginal fringe surrounding the dorsum is covered in a glassy secretion. The life history of *L.lectularius* is similar to that of *L.eucalypti*. For details see Notes under *L.eucalypti*. One exception is that the galls of developing young females of *L.lectularius* are located on succulent young twigs and buds rather than on leaves. Mature females of *L.lectularius* were collected by JWB from *Eucalyptuscamaldulensis* at La Trobe, and on other hosts and localities in Victoria during a relatively short period (February 14 to 20, 1972). This suggests that *L.lectularius* may reproduce with more synchrony than *L.eucalypti*. Eggs from females of *L.lectularius* held in the laboratory by JWB began to hatch approximately two weeks after oviposition.

In an unpublished manuscript, JWB treated as a separate species some of the larger specimens of what we consider to be *L.lectularius*. He noted that these specimens closely resemble the type material of *L.lectularius* and that the first-instar nymphs were identical, but pointed out several differences: specifically, the larger females have longer setae, more tubular ducts, a larger anus, more expanded tibial apices, and more translucent pores on the hind legs. Each of these traits appears to be correlated with body size across *Lachnodius* species. Therefore, we have opted to interpret this as part of the phenotypic variation found within *L.lectularius*.

Maskell (1896) described the adult female, the second-instar female, and the first-instar nymph of this species. Apparently, his description was based on material sent to him from Victoria by Mr C French. Type material of this species consists of specimens on 6 unstained slides prepared by Maskell, one adult female in the USNM and eleven adult females mounted by JWB from specimens from two boxes of dried material in NZAC. The original Maskell slides are labelled “*Dactylopius lectularius*” with “*Dactylopius*” crossed out and “*Lachnodius*” written above it, and “1895 – W. M. M.” There are no locality or collector data on these slides. The dry material was labelled only “*Dactylopius lectularius* – Australia” but the boxes were lost (see explanation in Materials and Methods). Only one of the original Maskell slides contains an entire adult female. JWB labelled that specimen as the lectotype in 1972 but this action was not published until now (see below). Of the remaining slides (paralectotypes), one contains female mouthparts, one the posterior body and antenna of an adult female, one a second-instar female, and two contain first-instar nymphs. When JWB slide-mounted specimens from Maskell’s dry material in 1972, he labelled the slides with the collection data from the original description (but with the wrong collector name), rather than what was written on the box.

Concerning the type material of *L.lectularius*, Maskell (1896: 401) only stated that “Mr. French has sent me a number of specimens and says, ‘It does great damage to young trees at Mooroopna, Goulburn River, Victoria’.” Therefore, we assume that all of his material was from this one source. Specimens in the dry material are mostly parasitized mummies, and JWB only obtained two satisfactory slide preparations. Both of these adult females show evidence of having been parasitized, containing parasitoid mandibles, encyrtiform eggshells, and small sclerotized first-stage parasitoid larvae. Maskell (1896: 401) recorded the habitat from which the type material was derived as “In Australia, on *Eucalyptus rostrata*.” *Eucalyptusrostrata* is a junior synonym of *E.camaldulensis*, the “river red gum,” a common species throughout southeastern Australia (Chippendale 1988).

The adult female specimens of *L.lectularius* in the Maskell collection do not agree in all details with his published description and figures. We consider that the discrepancies are errors in Maskell’s interpretation. Morrison and Morrison (1922) noted that Maskell’s descriptions often were inaccurate. Here we point out the mismatches between his description and specimens. In his 1896 description, Maskell stated that the adult female has an “Epidermis bearing many short fine hairs, and near the cephalic and abdominal extremities are two curved series of stronger spiny hairs, about sixty in each.” His figure of the female abdomen (Maskell 1896: Plate XXI, fig. 16) shows a series of spine-like setae in the area behind the anal ring. In the drawing these are thicker and more conical in form than the fringe setae, which are depicted (Plate XXI, fig. 17) as being nearly digitiform. By contrast, the Maskell specimens do not have conical or spiniform setae posterior to the anal area, although many of the setae appear to have been broken off and look somewhat like stiff bristles. On the other hand, in fresh preparations, the ventral setae in this region are quite elongate. Thus, we think that he simply confused body surfaces. Maskell also refers to a pair of “strong short conical spines” set close together between the antennae in some specimens, but not in all. In this position in the lectotype, we found a pair of parasitoid mandibles, which he must have mistaken for spines. Likewise, he mistook several pairs of parasitoid mandibles for spines in his description of the second-instar female. Maskell counted 24 of these structures, which is consistent with JWB’s observation that the encyrtids that attack *Lachnodius* species can be highly gregarious.

In his notes, JWB recorded having studied two specimens that were not seen by PJG or NBH: Queensland: two adult females: *Eucalyptus* sp., gall no. 9, Acacia Ridge, Brisbane, 10 Jan1968, EC Dahms (these probably are housed in the Queensland Museum in Brisbane).

#### Material examined.

***Lectotype*** (here designated): **Victoria**: adult female: on slide labelled: “*Lachnodius* / *Dactylopius* / *lectularius* / adult female / 1895 W.M.M.” (ANIC). ***Paralectotypes***: **Victoria**: five slides: adult female mouth parts, adult female posterior body and antennae, one second-instar female, and two first-instar nymphs: same label data as lectotype (NZAC); eleven adult females, on six slides prepared and labelled by JW Beardsley from Maskell dry material: “VICTORIA / Mooroopna / Goulburn Riv. / ?1896 / W. W. Froggatt [SIC] / *Eucalyptus* / *rostrata* in / twig depression” (NZAC); one adult female, on slide labelled: “*Lachnodius* / *lectularius* / Mask. / Australia / Mask. Coll. No. 453” (USNM). Note that JWB made an error in writing the collector as “W.W. Froggatt”, as the original specimens were collected by C. French. Also, the dry material that JWB mounted did not bear the collection data that he put on his slide labels, but was added by JWB based on the data cited in Froggatt’s original description. ***Additional material***: **Australian Capital Territory**: one adult female, ex pit in swollen woody stems, *Eucalyptus* sp., Canberra, Black Mountain, Coll. 6 Dec 1996, JH Martin 6845 (ANIC). **New South Wales**: three adult females: *Eucalyptus* sp. (bloodwood), 5 km W of Bogangar, 23 Nov 1986, S Bhatti, PJG, and C Reid (ANIC); two adult females: ex pits in swollen stems, *E.dives*, 2 km S of Captain’s Flat, 35.58S, 149.47E, 4 Jan 2009, PJG (ANIC); one adult female: ex pit gall, *Eucalyptus* sp., Congo, 35.95S, 150.15E, 6 Jan 1992, PJG (ANIC); one adult female: ex swollen stem, *Eucalyptus* sp., 22 km NE of Griffith, Whitton Stock Route, 34.15S, 146.20E, 30 Oct 1993, PJG (ANIC); one adult female: *Eucalyptus* sp., E of Walcha, Oxley Highway, 31.21S, 151.90E, 1130 m, 25 May 2005, LG Cook, LGC00345, NH87 (ANIC); two adult females: ex depressions in swollen fruit, *Eucalyptus* sp., N. Sydney, Beacon Hill, Peninsula Views Estate, 18 Sep 1993, LG Cook, LachB (ANIC). **Queensland**: four adult females (all parasitized): *Eucalyptusdrepanophylla*, R-8 Doongul, 27 Sep 1939, AR Brimblecombe (QDPC); one adult female, three second-instar nymphs, 14 first-instar nymphs: *E.crebra*, Moggill, 20 Nov 1953, AR Brimblecombe (QDPC) (these three slides could not be located at QDPC). **South Australia**: eleven adult females, three second-instar females, one first-instar nymph: ex swellings on twigs or stems, *E.camaldulensis*, Glen Osmond, 6 Oct 1982, GS Taylor, HMB Specimen Index No. 20/82 (ANIC). **Victoria**: five adult females, seven first-instar nymphs: ex pits in twigs, *Eucalyptusradiata*, 20 miles [32 km] W of Drouin, 20 Feb 1972, JWB (BPBM except one slide of nymphs in ANIC); 14 first-instar nymphs: ex ovisac on bark, *E.camaldulensis*, Bundoora, La Trobe University, Coll. 21 Feb 1972, JWB (BPBM); three adult female: ex pits in twigs, *E.camaldulensis*, Bundoora, La Trobe University, Coll. 14 Feb 1972, JWB (BPBM); two adult females, one second-instar male: under bark, *E.camaldulensis*, Bundoora, La Trobe University, Wildlife Reserves, Ring Road, 37.72S, 145.05E, 14 Feb 2005, NBH and PJG, NH41, NH154, NH161 (ANIC); two adult females: ex pits in swollen stems, *E.viminalis*, Cranbourne, Royal Botanic Gardens Cranbourne, Possum Gully Track, 38.13S, 145.28E, 9 Feb 2005, PJG, NH40, NH115 (ANIC); one adult female: ex pit in swollen stem, *E.aromaphloia*, Grampians Nat. Park, Victoria Valley, Glenelg River Road, 37.23S, 142.41E, 6 Feb 2005, NBH and PJG, NH119 (ANIC); one adult female: ex pit in swollen stem, *E. ?polyanthemos*, Melbourne, North Warrandyte, corner of Overbank Road and Glynns Road, 37.73S, 145.20E, 14 Feb 2005, NBH and PJG, NH46 (ANIC); one second-instar female: ex pit in twig, *E.radiata*, Mt Eliza, 22 Feb 1972, JWB (BPBM); one adult female: in depression on swollen stem, *Eucalyptus* sp., near Hattah, Nov 1993, LG Cook (ANIC); five adult females, one second-instar female: ex pits in twigs, *E.viminalis*, Tooborac, 24 Feb 1972, JWB (BPBM); two adult females: ex pits in flower buds, *E.viminalis*, Tyabb, junction of Tooradin-Tyabb Road and Callanans Lane, 38.21S, 145.25E, 13 Feb 2005, NBH and PJG (NMV).

**Figure 7.**
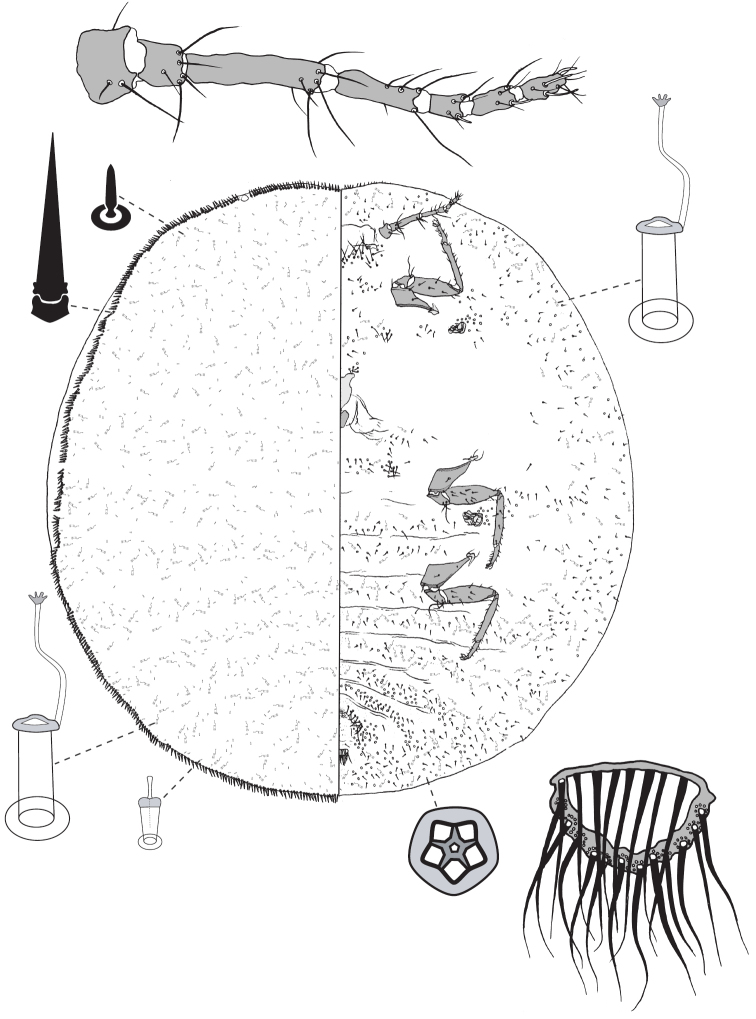
Adult female of *Lachnodiuslectularius* Maskell.

### Lachnodius maculosus

Taxon classificationAnimaliaHemipteraEriococcidae

Beardsley, Gullan & Hardy sp. n.

http://zoobank.org/182CD8B3-7EC4-45C5-9A27-9F61F902C4BB

Fig. 8

#### Diagnosis.

Dorsum with numerous sclerotic invaginations; marginal fringe of conical setae; some dorsal macrotubular ducts with seta touching rim; anal ring invaginated.

#### Description.

**Adult female** (n = 3). Body outline circular; length 3.45–4.84 mm (4.36 mm for holotype), greatest width 3.30–3.94 mm (3.94 mm for holotype). Eyes dorsal, very weakly developed, ca. 35 μm wide. Antennae seven-segmented; length 680–690 μm; with two hair-like setae on segment I, ca. three hair-like setae on segment II, two or three hair-like setae on segment III, three hair-like seta on segment IV, one fleshy seta on segment V, two hair-like setae + one fleshy seta on segment VI and six hair-like setae + three fleshy setae on segment V. Tentorial box with anterior extension of the dorsal arms, 285–335 μm long, 245–265 μm wide. Labium 125 μm long, 190–195 μm wide. Spiracles 130–155 μm long, 65–85 μm wide across atrium. Legs increasing in size caudad; fore legs: trochanter + femur 500 μm, tibia 425–460, tarsus 170–210 μm; mid legs: trochanter + femur 510–520 μm, tibia 445–480 μm, tarsus 170–210 μm; hind legs: trochanter + femur 555–560 μm, tibia 495–520 µm, tarsus 200–218 μm; claw 50–56 μm; fore coxa with 6 setae, mid and hind coxae each with 5 setae, trochanter with 5–7 setae, femur with 12–19 setae, tibia with 20–27 setae, tarsus with 6–15 setae; tarsal digitules 68–74 μm long, claw digitules 43–50 μm long; translucent pores on all segments of hind leg, ca. 60 pores on dorsal surface and ca. 30 pores on ventral surface. Anal ring invaginated, cuticle surrounding ring sclerotic, 68–78 μm wide, with 10–12 setae; ring setae 60–140 μm long. Pair of elongate caudal setae ca. 45 μm long.

*Dorsum*. Derm beset with sclerotic spicules (i.e., well-developed microtrichia), in addition to sclerotic varioles 8–12 μm wide. Dorsal setae lanceolate, 5–8 μm long, scattered over dorsum. Macrotubular ducts of two size-classes: (i) larger ducts ca. 20 μm long, with rim of dermal orifice ca. 10 μm wide; (ii) smaller ducts ca. 10 μm long, with rim of dermal orifice ca. 7 μm in diameter; many of larger ducts with one seta affixed to rim of dermal orifice. Microtubular ducts each ca. 7 μm long, with rim of dermal orifice ca. 2 μm wide, scattered over dorsum. Dorsum delimited by fringe of ca. 275 setae on each side of body; each seta with acute apex, length of setae 18–33 μm; marginal fringe interrupted between thorax and abdomen.

*Venter*. Ventral setae 22–60 μm long; elongate setae medial of each coxa decreasing in size caudad: ca. 100 μm long near fore coxa, ca. 55 μm long near hind coxa; longest setae on head 120–140 μm long. Macrotubular ducts of two types: (i) larger ducts with shaft subtending vestibule constricted, each ca. 22 μm long, with rim of dermal orifice ca. 6 μm wide, found along posterior margin and in transverse band across abdominal segment IV; (ii) smaller ducts with uniform shaft diameter ca. 15 μm long, with rim of dermal orifice ca. 4 μm wide, along margin anterior of larger ducts, in transverse rows across abdominal segments, amongst clusters of setae medial of each coxa. Quinquelocular pores of two distinct size-classes: (i) larger pores 5–6 μm in diameter, found on posterior abdominal segments; and (ii) smaller pores 3–4 μm in diameter, near spiracles and along margin.

#### Etymology.

The species name is taken from the Latin noun *macula* meaning spot, referring to the shallow, sclerotic pits on the dorsal body surface, combined with the Latin suffix -*osus* to give the meaning abundance of spots or spotted. The species epithet is a Latin masculine adjective.

#### Notes.

Adult females of *L.maculosus* are most similar those of *L.melliodorae* and *L.parathrix*. See notes under *L.melliodorae* for a comparison. Adult females of *L.maculosus* can be distinguished by having (i) two size classes of macrotubular duct on both the dorsal and ventral body surfaces (*L.melliodorae* and *L.parathrix* have only one size class per body surface); and (ii) numerous minute sclerotic invaginations on the dorsum, each with interior margin sinusoidal (*L.parathrix* without minute sclerotic invaginations, *L.melliodorae* with minute sclerotic invaginations urn-shaped, interior margin convex).

HMB’s Specimen Index card for collection 161/54 notes that the adult females were laying eggs in large numbers under the bark.

#### Material examined.

***Holotype***: **South Australia**: adult female, on slide: under bark of *Eucalyptus* sp., National Park, Belair, 5 Dec 1954, DC Swan, HMB Specimen Index No. 161/54 (ANIC). ***Paratypes***: **South Australia**: two adult females, same data as holotype (ANIC).

**Figure 8.**
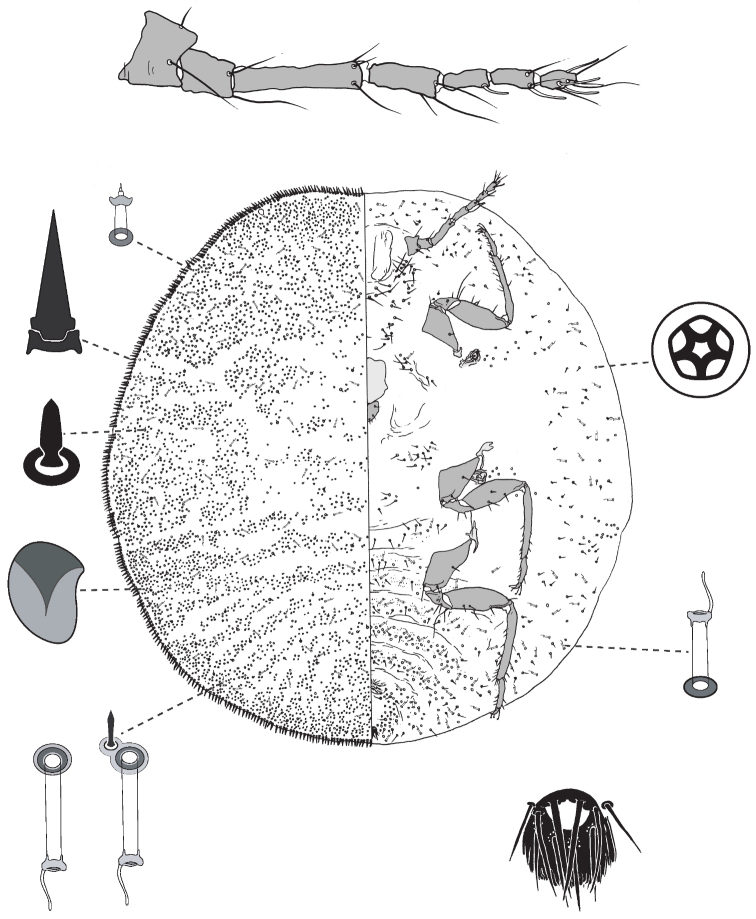
Adult female of *Lachnodiusmaculosus* sp. n.

### Lachnodius melliodorae

Taxon classificationAnimaliaHemipteraEriococcidae

Beardsley, Gullan & Hardy sp. n.

http://zoobank.org/E160E86A-DB9D-494E-A1B9-A2B019F16EC7

Fig. 9

#### Diagnosis.

Dorsum with numerous urn-shaped sclerotic invaginations; marginal fringe of curved setae; some dorsal macrotubular ducts with seta touching rim; anal ring invaginated.

#### Description.

**Adult female** (n = 15). Body outline circular to ovate; length 1.96–5.74 mm (4.02 mm for holotype), greatest width 1.53–3.90 mm (3.64 mm for holotype). Eyes dorsal, 38–45 μm wide. Antennae seven-segmented; length 680–882 μm; with 4–11 hair-like setae on segment I, 6–9 hair-like setae on segment II, 8–11 hair-like setae on segment III, 7–8 hair-like seta on segment IV, 3–5 hair-like setae + one fleshy seta on segment V, 5–6 hair-like setae + one fleshy seta on segment VI and six hair-like setae + three fleshy setae on segment V. Tentorial box with anterior extension of the dorsal arms, 350–475 μm long, 135–165 μm wide. Labium 135–165 μm long, 165–200 μm wide. Spiracles 140–225 μm long, 80–135 μm wide across atrium. Legs increasing in size caudad; fore legs: trochanter + femur 505–690 μm, tibia 410–560 µm, tarsus 183–250 μm; mid legs: trochanter + femur 548–750 μm, tibia 410–570 μm, tarsus 195–265 μm; hind legs: trochanter + femur 540–790 μm, tibia 490–590 µm, tarsus 200–263 μm; claw 45–63 μm; fore coxa with six setae, mid and hind coxae each with five setae, trochanter with 8–15 setae, femur with 20–37 setae, tibia with 28–50 setae, tarsus with 11–21 setae; tarsal digitules 73–93 μm long, claw digitules 45–68 μm long; translucent pores on all segments of hind leg, ca. 150 pores on dorsal surface and ca. 90 pores on ventral surface. Anal ring invaginated, cuticle surrounding ring sclerotic, 58–108 μm wide, with 10–15 setae; ring setae 45−108 μm long. Pair of elongate caudal setae usually absent, present in one specimen, ca. 28 μm long.

*Dorsum*. Derm beset with sclerotic spicules (i.e., well-developed microtrichia), in addition to sclerotic urns, each 4–6 μm wide. Dorsal setae lanceolate, 5–6 μm long, scattered over dorsum. Macrotubular ducts 15–20 μm long, with rim of dermal orifice 3–7 μm wide, ducts diminishing in size cephalad, many ducts with one seta affixed to rim of dermal orifice. Microtubular ducts ca. 5 μm long, with rim of dermal orifice ca. 2 μm wide, scattered over dorsum. Dorsum delimited by fringe of ca. 225 setae on each side of body; each seta slender and recurved, 15–23 μm long; marginal fringe interrupted by U-shaped sclerite between thorax and abdomen.

*Venter*. Ventral setae 20–50 μm long; elongate setae medial of each coxa decreasing in size caudad: 125–165 μm long near fore coxa, 50–95 μm long near hind coxa; longest setae on head 153–200 μm long. Macrotubular ducts each ca. 15 μm long, with rim of dermal orifice ca. 6 μm wide, found along margin and in a transverse row across each abdominal segment. Quinquelocular pores of two distinct size-classes: (i) larger pores ca. 5.5 μm in diameter, found on posterior abdominal segments; and (ii) smaller pores ca. 4 μm in diameter, near spiracles and along margin.

**Notes.** Adult females of *L.melliodorae* are most similar those of *L.parathrix* and *L.maculosus*. These three species share (i) a marginal fringe composed of close-set setae interrupted between the thorax and abdomen; (ii) two distinct size classes of quinquelocular pores on the venter; and (iii) several dorsal macrotubular ducts with a seta affixed to the dermal orifice. Adult females of *L.melliodorae* can be distinguished from those of *L.parathrix* and *L.maculosus* by having (i) recurved marginal setae (marginal setae straight in *L.parathrix* and *L.maculosus*) and (ii) an U-shaped sclerite between the thorax and abdomen on the margin on each side of the body (sclerite absent in *L.parathrix* and *L.maculosus*).

Young adult females collected near Benalla, Victoria, by PJG in 1996 and 1997 were pale yellow in life with dorsomedial longitudinal stripe of red-wine color; the anterior spiracular furrow was visible as a pale line on each side of the body. The dorsum was naked (no secretion), but each seta in the marginal fringe was covered in a glassy secretion. The second-instar female had a salmon-colored dorsum.

The type series is restricted to specimens collected at Lower Plenty in Victoria, where JWB made several collections of all instars of this species.

**First-instar nymph** (n = 8). Body outline ovate; length 280–360 μm, greatest width 215–290 μm. Eyes 12–15 µm wide. Antennae six-segmented but appearing 5-segmented due to partial fusion of segments III and IV; length 65–91 µm; with three hair-like setae on segment I, two hair-like setae on segment II, two hair-like setae on segment III, one fleshy seta on segment IV, one fleshy seta + two hair-like setae on segment V, three fleshy setae + six hair-like setae on segment VI. Tentorial box 63–68 µm long, 50–58 µm wide. Labium 20–25 µm long, 30–33 μm wide. Spiracles ca. 15 µm long, ca. 7 µm wide across atrium. Legs: trochanter + femur 68–75 µm, tibia 30–40 μm, tarsus 43–53 µm; claw 12–14 µm; coxa with ca. six setae, trochanter with four setae, femur with five setae, tibia with four setae, tarsus with five or six setae; tarsal digitules unequal, large proximal digitule 25–32 µm long, small distal digitule ca. 20 μm long, claw digitules 13–15 µm long. Anal ring 17 µm wide, with six setae, each seta ca. 20 μm long. Pair of elongate caudal setae 143–163 µm long.

*Dorsum*. Derm beset with weakly sclerotic spots, each 2–5 µm in greatest dimension. Dorsal setae ca. 5 µm long; two longitudinal rows on each side of body, medial row with two setae on prothorax, and one seta on each segment from mesothorax to abdominal segment VII, submedial row with three setae on prothorax and one seta on each segment from mesothorax to abdominal segment I. Microtubular ducts each ca. 5 μm long, with rim of dermal orifice subelliptical, ca. 2 μm wide and 3 μm long, two longitudinal rows on each side of body, medial row with one duct on each segment from head to metathorax + abdominal segment VIII, and submarginal row with two ducts on prothorax, one duct on each thoracic segment and abdominal segments I and V (Note: it was an arbitrary decision to include the ducts on each side of head and abdominal segment VIII in the “medial” row). Dorsum delimited by fringe of 31–34 setae on each side of body (excluding caudal setae), each seta 9–15 μm long and 7–9 µm width at base above socket, deltate, with anterolateral margin sinusoidal and posteromedial margin straight; weakly sclerotic cuticle surrounding each setal socket, these sclerotic areas coalescing around the three most posterior fringe setae and the caudal seta, forming broad caudal sclerotization.

*Venter*. Ventral setae 2–5 µm long, in three longitudinal rows on abdomen; one elongate (ca. 18 μm long) seta medial of each coxa, three elongate setae (18–27 μm long) in longitudinal row on each side of head. Multilocular pores trilocular, 5 µm in diameter; one near each spiracle. Ventral lobe seta absent.

**Notes.** The first-instar nymphs of *L.melliodorae* are most similar to those of *L.froggatti* and *L.lectularius*, which also have (i) dorsal sclerotic spots (poorly developed in *L.froggatti*); (ii) stout marginal setae subtended by patches of sclerotic cuticle; and (iii) broad caudal sclerotizations (but in *L.lectularius*, only the caudal seta and 1 enlarged fringe seta are part of the sclerotization). The first-instar nymphs of *L.eucalypti* lack both the sclerotized area surrounding the socket of each marginal seta and the broad caudal sclerotizations, but have dorsal sclerotic spots. The nymphs of all four species have a similar arrangement of ducts, pores and setae. The first-instar nymphs of *L.melliodorae* can be distinguished from those of *L.eucalypti*, *L.froggatti* and *L.lectularius* by the distinctive shape of the marginal setae: deltate, with anterolateral margin sinusoidal and posteromedial margin straight, and base broad (marginal setae of *L.eucalypti* mostly falcate, with setal base constricted; of *L.froggatti* falcate but shorter than those of *L.eucalypti*; of *L.lectularius* more elongate, conical, with both margins straight).

#### Etymology.

The species name refers to the name of the host from which the type material was collected. The species epithet is in the genitive singular.

#### Material examined.

***Holotype***: **Victoria**: adult female, on slide: ex open pit gall on twig, *Eucalyptusmelliodora*, Lower Plenty, 19 Dec 1971, JWB (ANIC). ***Paratypes***: **Victoria**: two adult females, same data as holotype (ANIC); four adult females, three second-instar females, same data as holotype, except V-241, 12 Dec 1971 (BPBM); one parasitized second-instar female, same data as holotype, except 16 Oct 1971; four adult females, one second-instar female and eight first-instar nymphs, same data as holotype except 1 Jan 1972 (first-instar nymphs reared in lab from ovisac) (BPBM except one slide of nymphs in ANIC).

Additional material: two adult females, two second-instar females with pharate adults: ex pits on stems, *Eucalyptus* sp. sapling, 10 km NNW of Benalla, roadside, 36.48S 145.95E, 22 Jun 1996 and 25 Apr 1997, PJG (ANIC). **New South Wales**: one adult female: ex open pit twig gall, *Eucalyptus* sp., 15 miles [24 km] S of Kempsey, Pacific Highway, 26 Mar 1972, JWB (BPBM). **South Australia**: one adult female: under bark of *E.viminalis*, 5 km S of Mylor, Mt Lofty Ranges, 18 Nov 1979 MS Harvey and D Cukier (ANIC).

**Figure 9.**
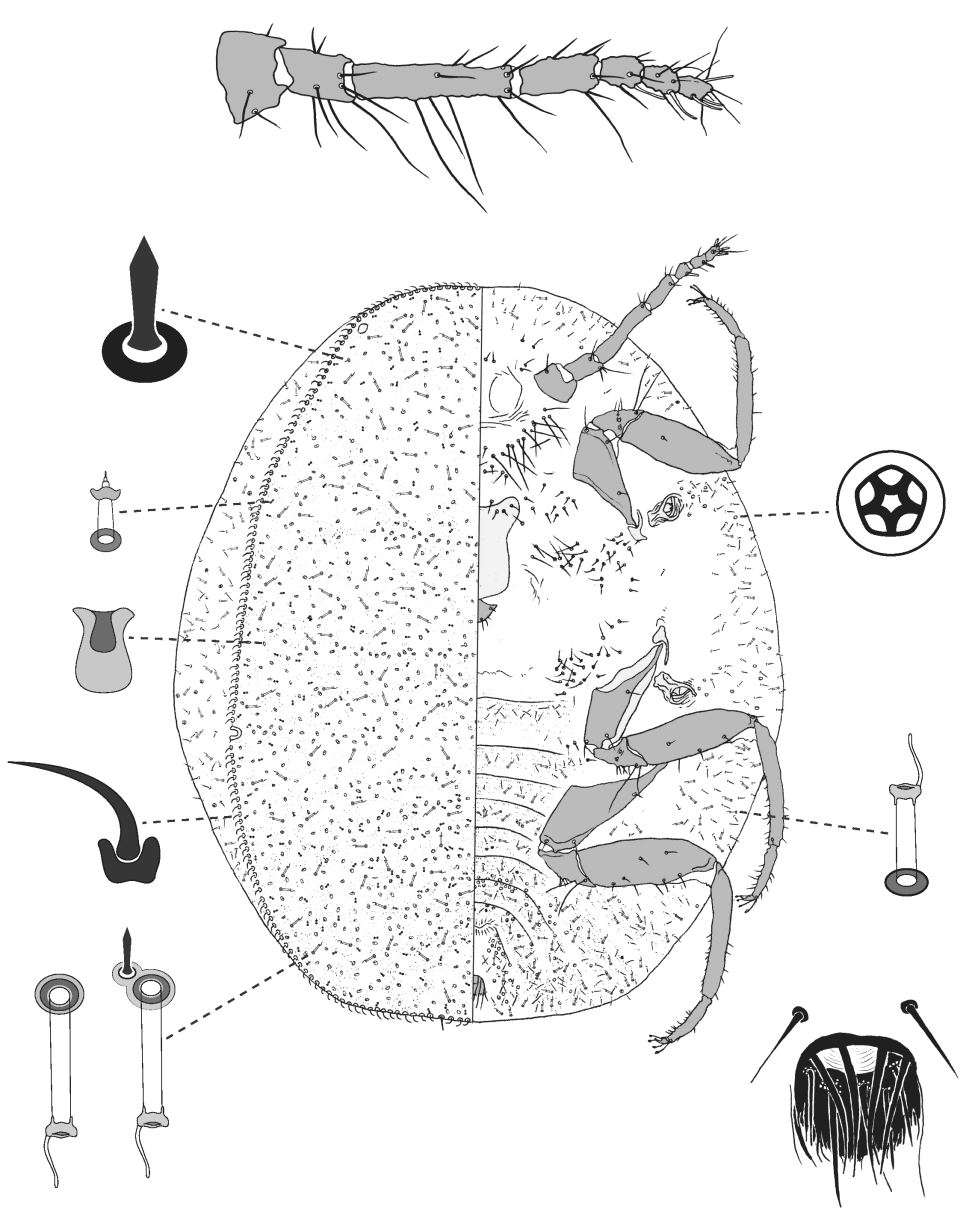
Adult female of *Lachnodiusmelliodorae* sp. n.

### Lachnodius newi

Taxon classificationAnimaliaHemipteraEriococcidae

Beardsley, Gullan & Hardy sp. n.

http://zoobank.org/83A4BFEC-226E-4382-8989-F674F704A2B3

Fig. 10

#### Diagnosis.

Dorsum without sclerotic invaginations; marginal fringe of curved setae; anal ring invaginated.

#### Description.

**Adult female** (n = 1). Body outline of holotype slightly oblong; length 1.98 mm, greatest width 1.35 mm. Eyes 33 μm wide. Antennae seven-segmented; length 490 μm; with seven hair-like setae on segment I, ca. four hair-like setae on segment II, ten hair-like setae on segment III, four hair-like seta on segment IV, two hair-like setae + one fleshy seta on segment V, three hair-like setae + one fleshy seta on segment VI and six hair-like setae + three fleshy setae on segment V. Tentorial box with anterior extension of the dorsal arms, 245 μm long, 168 μm wide. Labium 98 μm long, 123 μm wide. Spiracles 115–130 μm long, 75–82 μm wide across atrium. Legs increasing in size caudad; fore legs: trochanter + femur 360 μm, tibia 340 µm, tarsus 130 μm; mid legs: trochanter + femur 385 μm, tibia 350 μm, tarsus 140 μm; hind legs: trochanter + femur 390 μm, tibia 335 µm, tarsus 133 μm; claw 38–40 μm; fore coxa with six setae, mid and hind coxae each with five setae, trochanter with four setae, femur with ca. 12 setae, tibia with 14–16 setae, tarsus with ten or eleven setae; tarsal digitules 63–70 μm long, claw digitules 48 μm long; translucent pores on all segments of hind leg. Anal ring invaginated, cuticle surrounding ring sclerotic, 78 μm wide, with 12 setae; ring setae 63–75 μm long. Pair of elongate caudal setae absent.

*Dorsum*. Derm densely covered with sclerotic spicules (i.e., well-developed microtrichia). Sclerotic urns and varioles absent. Dorsal setae ca. 3 μm long, scattered over dorsum. Macrotubular ducts ca. 10 μm long, with rim of dermal orifice ca. 5 μm wide. Microtubular ducts ca. 5 μm long, with rim of dermal orifice ca. 2 μm wide, scattered over dorsum. Dorsum delimited by fringe of ca. 180 setae on each side of body; each seta slender and recurved, length of setae ca. 13 μm, each setal socket surrounded by irregular patch of sclerotic cuticle.

*Venter*. Ventral setae 10–40 μm long; elongate setae medial of each coxa decreasing in size caudad: ca. 68 μm long near fore coxa, ca. 45 μm long near hind coxa; longest setae on head ca. 105 μm long. Macrotubular ducts each ca. 15 μm long, with rim of dermal orifice ca. 5 μm wide, found along margin and in transverse band across each abdominal segment. Quinquelocular pores 5 μm in diameter, dense on posterior abdominal segments, clustered around spiracles.

#### Etymology.

This species is dedicated to Dr TR New, of the former Department of Zoology (now Ecology, Environment and Evolution), La Trobe University, who accompanied JWB during many collecting trips made in Victoria during 1971–72, and who guided JWB to the spot where this species was discovered. The species epithet is a noun in the genitive singular.

#### Notes.

The holotype is the only specimen known for this species, but it is distinctive. The specimen is relatively small and probably not fully expanded. However, the modest size of the antennae and legs, in comparison with other twig gall-inhabiting species (e.g., *L.lectularius*) suggests that even fully expanded adults of *L.newi* would not measure much more than 4 mm long. The adult female of *L.newi* would be confused most easily with those of *L.melliodorae*, as both species have strongly recurved marginal setae. The adult female of *L.newi* can be distinguished from those of *L.melliodorae* by lacking urn-shaped sclerotic structures on the dorsum (present in *L.melliodorae*). In their place are heavily sclerotized microtrichia. The adult female of *L.newi* can be further differentiated from those of *L.melliodorae* by having no dorsal macrotubular ducts with a seta touching the rim of the dermal orifice (some present in *L.melliodorae*) and by lacking long setae at the middle of the posterolateral edge of antennal segment III.

#### Material examined.

***Holotype***: **Victoria**: adult female, on slide: ex shallow pit gall in twig, *Eucalyptusviminalis*, Otway Ranges, Parker Road, heath area, 27 Oct 1971 JWB, V-168 (ANIC).

**Figure 10.**
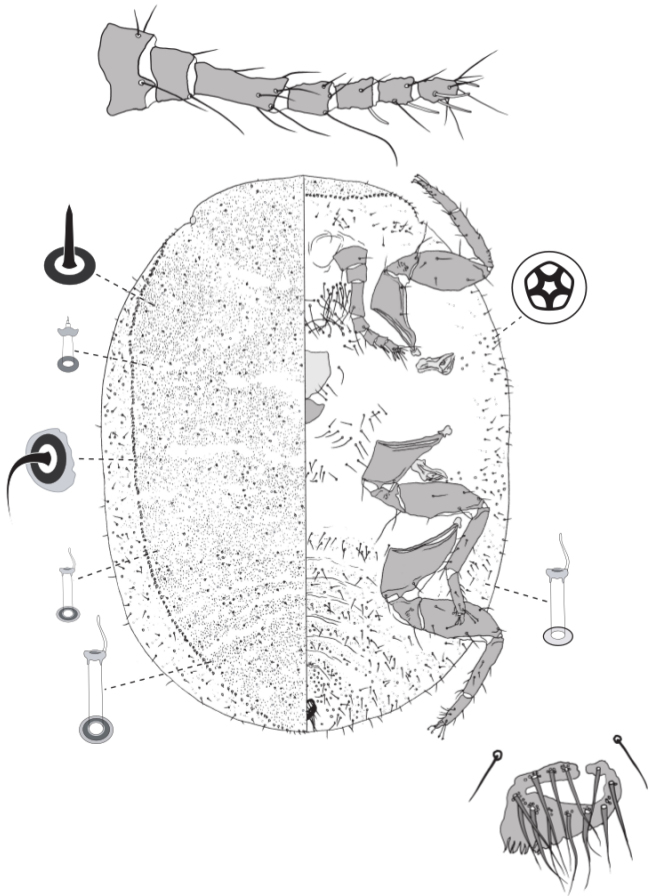
Adult female of *Lachnodiusnewi* sp. n.

### Lachnodius parathrix

Taxon classificationAnimaliaHemipteraEriococcidae

Beardsley, Gullan & Hardy sp. n.

http://zoobank.org/D3B700AC-23F7-47EF-9C94-DCF5F4C27538

Figs 2c , 11

#### Diagnosis.

Dorsum without sclerotic invaginations; marginal fringe of conical setae; some dorsal macrotubular ducts with seta touching rim; anal ring invaginated.

#### Description.

**Adult female** (n = 3). Body outline circular; length 1.60–2.70 mm (2.70 mm for holotype), greatest width 1.50–2.22 mm (2.08 mm for holotype). Eyes 25–40 μm wide. Antennae seven-segmented; length 405–700 μm; with six hair-like setae on segment I, six hair-like setae on segment II, 3–6 hair-like setae on segment III, four hair-like seta on segment IV, one fleshy seta on segment V, two hair-like setae + one fleshy seta on segment VI and six hair-like setae + three fleshy setae on segment V. Tentorial box with anterior extension of the dorsal arms, 150–210 μm long, 125–170 μm wide. Labium 90–110 μm long, 75–110 μm wide. Spiracles 88–110 μm long, 45–70 μm wide across atrium. Legs increasing in size caudad; fore legs: trochanter + femur 280–540 μm, tibia 210−420 µm, tarsus 120–170 μm; mid legs: trochanter + femur 290–560 μm, tibia 210–420 μm, tarsus 120–180 μm; hind legs: trochanter + femur 320–610 μm, tibia 245–470 µm, tarsus 125–180 μm; claw 30–45 μm; coxa with six setae, trochanter with 5–7 setae, femur with 10–16 setae, tibia with 18–26 setae, tarsus with 9–12 setae; tarsal digitules 50–70 μm long, claw digitules 30–50 μm long; translucent pores on all segments of hind leg (except coxae of one female), ca. 80 pores on dorsal surface and ca. 50 pores on ventral surface. Anal ring invaginated, cuticle surrounding ring sclerotic, 63–77 μm wide, with 12–16 setae; ring setae 35–90 μm long. Pair of elongate caudal setae absent.

*Dorsum*. Derm beset with sclerotic spicules (i.e., well-developed microtrichia). Sclerotic urns and varioles absent. Dorsal setae lanceolate, 3–5 μm long, sparsely scattered over dorsum. Macrotubular ducts 10–12 μm long, with rim of dermal orifice ca. 5 μm wide; many of larger ducts with one seta affixed to rim of dermal orifice. Microtubular ducts ca. 4 μm long, with rim of dermal orifice ca. 2 μm wide, scattered over dorsum. Dorsum delimited by fringe of 210–250 setae on each side of body; each seta with acute apex, length of setae 20–35 μm; marginal fringe interrupted between thorax and abdomen.

*Venter*. Ventral setae 10–35 μm long; elongate setae medial of each coxa (50–110 μm long), and in a transverse band posterior of frontal lobes (longest seta 105–135 μm long). Macrotubular ducts each 10–15 μm long, with rim of dermal orifice 3–4 μm wide, found along margin and in transverse band across each abdominal segment. Quinquelocular pores of two distinct size-classes: (i) larger pores 5–6 μm in diameter, found on posterior abdominal segments; and (ii) smaller pores ca. 3 μm in diameter, near spiracles and along margin.

**Second-instar female** (n = 1). Broadly oval, length 2.0 mm. Antennae 6-segmented, short (160–190 µm total length), tapering base to apex, apical segment longest. Legs short, broad, tibiae and tarsi fused, claws weakly developed. Anal ring ca. 40 µm wide, with 10 setae each ca. 25 µm maximum length; a pair of setae, 27–30 µm long, just anterior to anal ring. Dorsum with sparse, small, peg-like setae ca. 4–8 µm long; tubular ducts apparently absent. Marginal fringe with ca. 140 conical setae, each 35–45 µm long, on each side of body. Ventral setae very sparse, filiform, up to 15 µm long; tubular ducts absent; with a widely spaces series of quinquelocular pores, each 3–4 µm in diameter, just inside the marginal fringe and a very few on thorax between margin and spiracles.

#### Etymology.

The species name is based on the Greek words *para*, meaning near or beside, and *thrix*, meaning hair, referring to the close-set setae forming the marginal fringe. It is a noun in apposition.

#### Notes.

The description of the adult female is based on specimens from both Victoria and New South Wales because they agree in all diagnostic features. The adult female from near Narooma has longer antennae and leg segments and appears to have no translucent pores on the hind coxae, compared with the two Victoria females, but this variation may be due to differences in nutrition or developmental temperatures. Adult females of *L.parathrix* are most similar those of *L.melliodorae* and *L.maculosus*, but can be easily distinguished by lacking the peculiar dorsal urns and varioles present in those species. The two known host species of *L.parathrix* belong to *Eucalyptus* series *Radiatae* and are characterized by the juvenile leaves having numerous oil glands (Brooker 2000).

#### Material examined.

***Holotype***: **Victoria**: adult female, on slide: ex open pit gall on young twig, *Eucalyptusradiata*, 15 miles [24 km] W of Drouin, Princess Highway, 23 Jan 1972, JWB (ANIC). ***Paratypes***: **Victoria**: one adult female, same data as holotype (BPBM); one pre-adult female exuviae, same data as holotype except: 20 miles [32 km] W of Drouin, 30 Jan 1972 (BPBM). **New South Wales**: one adult female: ex pit on midrib of leaf, *E.elata*, ca. 8 km WNW of Narooma, Wagonga Scenic Drive, 36.20S, 150.05E, 31 Dec 2008, PJG (ANIC).

**Figure 11.**
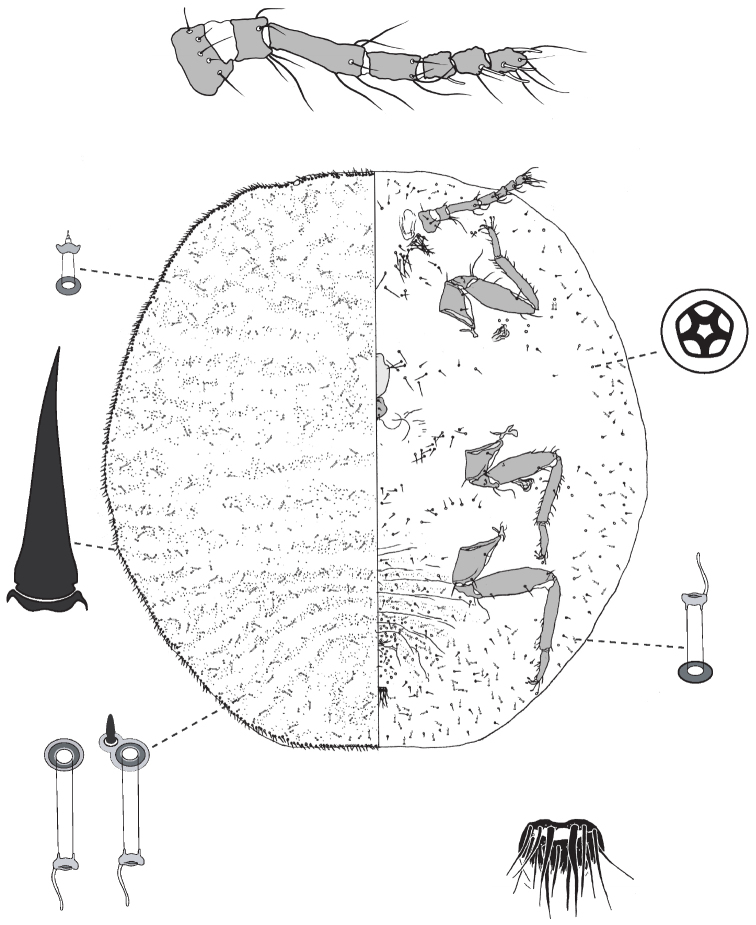
Adult female of *Lachnodiusparathrix* sp. n.

### Lachnodius sealakeensis

Taxon classificationAnimaliaHemipteraEriococcidae

Gullan & Hardy sp. n.

http://zoobank.org/2FCC872E-35E0-4BCF-88F1-FC47316422B5

Figs 2d , 12

#### Diagnosis.

Dorsum with dermal orifice of each microtubular duct surrounded by sclerosis; marginal fringe of truncate setae; dorsal setae capitate; anal ring invaginated.

#### Description.

**Adult female** (n = 16). Body outline oval; length 0.84–1.45 mm (1.18 mm for holotype), greatest width 0.70−1.28 mm (0.90 mm for holotype). Eyes not apparent. Antennae seven-segmented; length 230–355 μm; with two hair-like setae on segment I, one hair-like seta on segment II, one hair-like seta on segment III, two hair-like seta on segment IV, one fleshy seta on segment V, two hair-like setae + one fleshy seta on segment VI and six hair-like setae + three fleshy setae on segment V. Tentorial box with anterior extension of the dorsal arms, 138–190 μm long, 123–155 μm wide. Labium 60–75 μm long, 60–100 μm wide. Spiracles 45–68 μm long, 25–35 μm wide across atrium. Legs increasing in size caudad; fore legs: trochanter + femur 165–250 μm, tibia 105–175, tarsus 80–125 μm; mid legs: trochanter + femur 165−263 μm, tibia 105–165 μm, tarsus 90–125 μm; hind legs: trochanter + femur 190–275 μm, tibia 110–175, tarsus 85–125 μm; claw 28–38 μm; fore coxa with six setae, mid and hind coxae each with five setae, trochanter with four setae, femur with 6–8 setae, tibia with 6–8 setae, tarsus with 7–9 setae; tarsal digitules 45–60 μm long, claw digitules 25–40 μm long; translucent pores on all segments of hind leg. Anal ring invaginated, cuticle surrounding ring sclerotic, 38–75 μm wide, with 10–12 setae; ring setae 40–73 μm long. Pair of elongate caudal setae absent.

*Dorsum*. Derm covered with sclerotic spicules (i.e., well-developed acanthae or microtrichia). Sclerotic urns and varioles absent but dermal orifice of each microtubular duct surrounded by sclerotic region. Dorsal setae capitate 5–7 μm long, scattered over dorsum. Macrotubular ducts absent. Microtubular ducts ca. 5 μm long, with oral rim ca. 2 μm wide, scattered over dorsum. Dorsum delimited by fringe of 110–150 setae on each side of body, each seta subconical, most setae with truncate, serrated apices, a few setae with acute apices, length of setae 25–45 μm.

*Venter*. Ventral setae 10–30 (mostly 20–25) μm long; elongate setae medial of each coxa decreasing in size caudad: 50–80 μm long near fore coxa, 25–30 μm long near hind coxa; longest setae on head 63–123 μm long. Macrotubular ducts each ca. 15 μm long, with oral rim ca. 5 μm wide, duct shaft subtending vestibule constricted; in transverse band across each abdominal segment. Quinquelocular pores of one size-classes: 4–5 μm in diameter, on posterior abdominal segments and around margin, small clusters around spiracles.

#### Etymology.

The species name refers to the type locality, Sea Lake, Victoria. The name is an adjective with the suffix derived from the Latin -*ensis*, denoting place or locality.

#### Notes.

Adult females of *L.sealakeensis* are most superficially similar to those of *L.maculosus*; both species occur under bark, have an invaginated anus, and relatively robust, subconical marginal setae. Adult females of *L.sealakeensis* can be readily distinguished from those of *L.maculosus* by (i) the lack of dorsal macrotubular ducts (two size-classes present in *L.maculosus*); (ii) dorsal microtubular ducts with sclerotic surrounds (these are unique among *Sphaerococcopsis* and *Lachnodius* spp.); (iii) capitate dorsal setae (lanceolate in *L.maculosus*); and (iv) the ventral surface of abdomen with macrotubular ducts with the shaft constricted distally (not constricted distally in *L.maculosus*). Capitate dorsal setae like those of *L.sealakeensis* are also present on adult female of *Sphaerococcopsisplatynotum* Beardsley and *S.umbilicus* Beardsley. These differ from *L.sealakeensis* in having (i) 6-segmented antennae (7-segmented in *L.sealakeensis*); (ii) hind legs much larger than fore and mid legs (all legs subequal in *L.sealakeensis*); and (iii) venter much larger than dorsum (venter and dorsum subequal in *L.sealakeensi*).

This is the only species treated here that was not part of JWB’s view of *Lachnodius* since the only known specimens were not collected until after his death. NBH and PJG share authorship of its name.

#### Material examined.

***Holotype***: **Victoria**: adult female, on slide: ex pit under loose bark of *Eucalyptusoleosa*, ca. 6 km N of Sea Lake, intersection of Lake Tyrell Road and Calder Highway, 35.45S, 142.83E, NBH and PJG, 5 Feb 2005 (ANIC). ***Paratypes***: **Victoria**: 17 adult females (all on separate slides), same data as holotype, one female is DNA voucher NH47 (ANIC except 2 slides in NMV; also 2 slides deposited in QDPC in 2009 but could not be located in 2018).

**Figure 12.**
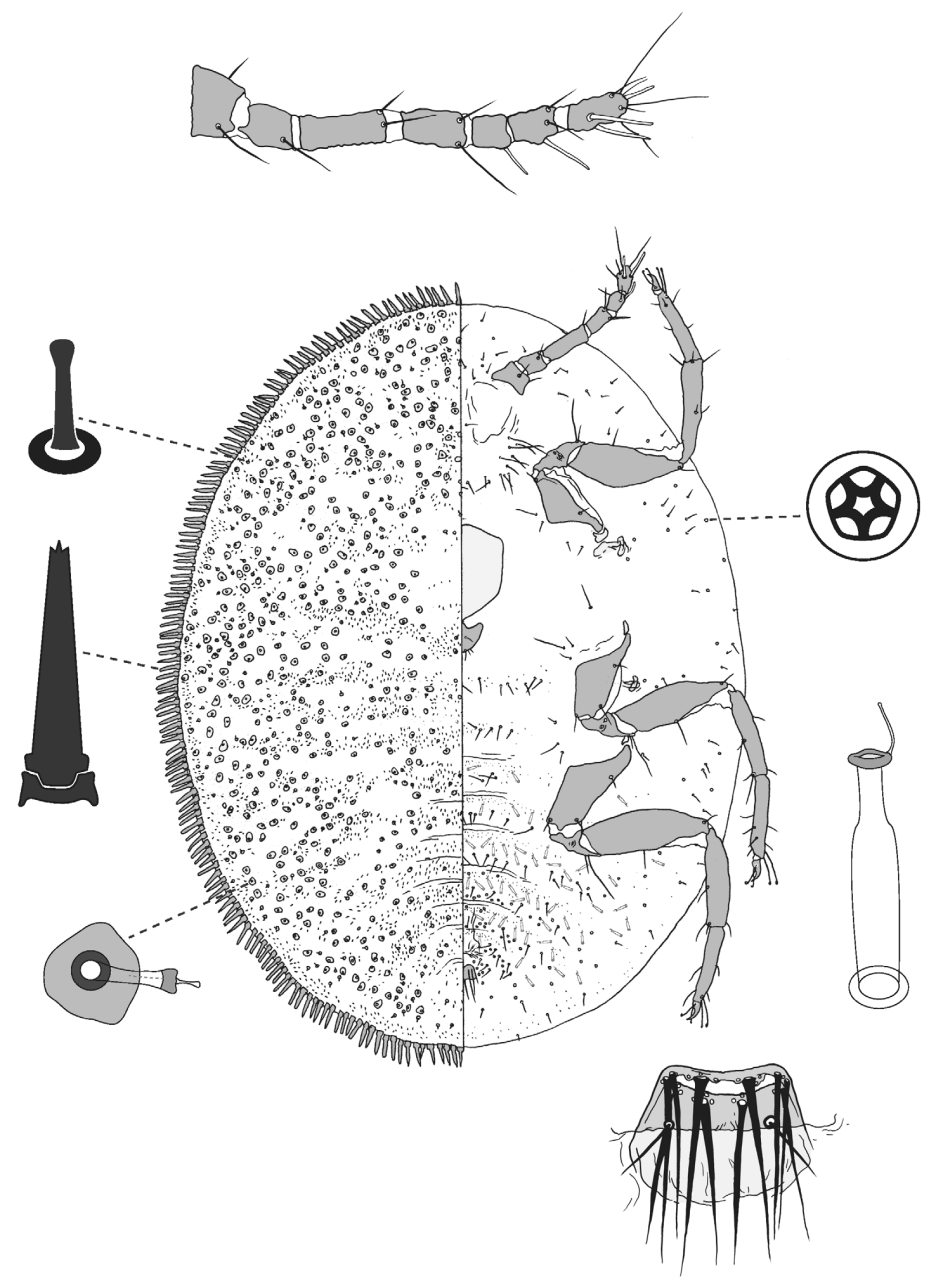
Adult female of *Lachnodiussealakeensis* sp. n.

## Discussion

This taxonomic work was begun in 1971 by the late Dr JW Beardsley, while he was a visiting Fulbright Research Scholar in the then Zoology Department, La Trobe University, Bundoora, Victoria. During that period, Beardsley encountered a number of undescribed taxa that he felt were new species of *Lachnodius* Maskell. Subsequently, he borrowed specimens of *Lachnodius* from Australian collections, especially those made by the late Ms HM Brookes (formerly of the Waite Agricultural Research Institute of the University of Adelaide) and PJG. He also visited the New Zealand Arthropod Collection in Auckland to examine Maskell’s type material. The demands of other work, including ten years as the Chair of the Department of Entomology at the University of Hawaii, delayed progress on his *Lachnodius* revision. That delay ended with his retirement, and by 1993, he had completed written descriptions for several species. However, arthritis in his hands kept him from being able to illustrate them and, when Beardsley died suddenly on 5 February 2001 (Anwar 2001), his work on *Lachnodius* was still unpublished. At that point, PJG acquired Beardsley’s notes and slides, with the intention of completing his work, an effort that NBH joined.

When PJG and NBH took over, they modified Beardsley’s taxonomic concepts. Specifically, of the species he included in his unpublished revision of *Lachnodius*, they described one as a species of *Opisthoscelis* (Hardy and Gullan 2010), one as the sole member of the genus *Heathcotia* Hardy & Beardsley (Hardy et al. 2011), and six as species of *Lobimargo* Hardy & Gullan (Hardy et al. 2011). This paper treats what is left of Beardsley’s concept of *Lachnodius*, and completes his revisionary work. Nevertheless, the monophyly of what we have left of Beardsley’s concept of *Lachnodius* is uncertain. These species clearly are closely related to *Sphaerococcopsis*, as well as to *Opisthoscelis* Schrader and *Tanyscelis* Hardy & Gullan. It could be that some are more closely related to these other genera than to some of the other species of *Lachnodius*. More phylogenetic work is required to resolve these relationships.

## Supplementary Material

XML Treatment for Lachnodius

XML Treatment for Lachnodius brimblecombei

XML Treatment for Lachnodius eucalypti

XML Treatment for Lachnodius froggatti

XML Treatment for Lachnodius hirsutus

XML Treatment for Lachnodius lectularius

XML Treatment for Lachnodius maculosus

XML Treatment for Lachnodius melliodorae

XML Treatment for Lachnodius newi

XML Treatment for Lachnodius parathrix

XML Treatment for Lachnodius sealakeensis

## References

[B1] Andersen AN (1989) Impact of insect predation on ovule survivorship in *Eucalyptusbaxteri*. Journal of Ecology 77: 62–69. 10.2307/2260916

[B2] Anwar Y (2001) John ‘Jack’ Beardsley Jr., Isle expert on insects, dead at 74. HonoluluAdvertiser.com, Wednesday, February 14, 2001. http://the.honoluluadvertiser.com/2001/Feb/14/214localnews26.html [accessed 17 July 2017]

[B3] Beardsley JW (1972) Systematics of Australian coccid genera *Lachnodius* Maskell and *Sphaerococcopsis* Cockerell (Homoptera). 14^th^ International Congress of Entomology Abstracts, 144.

[B4] Beardsley JW (1974) A review of the genus *Sphaerococcopsis* Cockerell, with descriptions of two new species (Homoptera: Coccoidea). Proceedings of the Hawaiian Entomological Society 21: 329–342.

[B5] Beardsley JW (1982) On the taxonomy of the genus *Pseudopsylla* Froggatt, with a redescription of the type species (Homoptera: Coccoidea). Proceedings of the Hawaiian Entomological Society 24: 31–35.

[B6] Brooker MIH (2000) A new classification of the genus *Eucalyptus* L’Hér. (Myrtaceae) Australian Systematic Botany 13: 79–148. 10.1071/SB98008

[B7] Brooker I (2002) Botany of the eucalypts. In: Coppen JJW (Ed.) Eucalyptus: The genus *Eucalyptus*. CRC Press, London, 464 pp 10.4324/9780203219430_chapter_1

[B8] Chippendale GM (1988) *Eucalyptus*, *Angophora* (Myrtaceae). In: George AS (Ed.) Flora of Australia 19. Australian Government Publishing Service, Canberra, 540 pp.

[B9] Cook LG Gullan PJ (2004) The gall-inducing habit has evolved multiple times among the eriococcid scale insects (Sternorrhyncha: Coccoidea: Eriococcidae). Biological Journal of the Linnean Society 83: 441–452. 10.1111/j.1095-8312.2004.00396.x

[B10] Cox JM Williams DJ (1987) Do the Eriococcidae form a monophyletic group? Bollettino del Laboratorio di Entomologia Agraria ’Filippo Silvestri’ 43: 13–17.

[B11] Deitz LL Tocker MF (1980) W. M. Maskell’s Homoptera: Species-group names and type material. New Zealand Department of Scientific and Industrial Research, Information Series 146: 1–76.

[B12] Fernald ME (1903) A catalogue of the Coccidae of the world. Bulletin of the Hatch Experiment Station of the Massachusetts Agricultural College 88: 1–360.

[B13] Ferris GF (1955) Some miscellaneous Coccoidea (Insecta: Homoptera). (Contribution No. 91). Microentomology 20: 22–29.

[B14] Froggatt WW (1917) A descriptive catalogue of the scale insects (“Coccidae”) of Australia. (Part II). Agricultural Gazette of New South Wales 28: 134–140.

[B15] Froggatt WW (1921) A descriptive catalogue of the scale insects (‘Coccidae’) of Australia. Part III. Department of Agriculture, New South Wales Science Bulletin 19: 1–43.

[B16] García Morales M Denno BD Miller DR Miller GL Ben-Dov Y Hardy NB (2016) ScaleNet: a literature-based model of scale insect biology and systematics. Database. 10.1093/database/bav118 http://scalenet.info [accessed 10 September 2018] PMC4747323 26861659

[B17] Gullan PJ (1984a) Froggatt’s accession notebooks. Australian Entomological Magazine 10: 91–92.

[B18] Gullan PJ (1984b) A revision of the gall-forming coccoid genus *Apiomorpha* Rübsaamen (Homoptera: Eriococcidae: Apiomorphinae). Australian Journal of Zoology Supplementary Series, No. 99, 203 pp.

[B19] Gullan PJ Miller DR Cook LG (2005) Gall-inducing scale insects (Hemiptera: Sternorrhyncha: Coccoidea). In: Raman A Schaefer CW Withers TM (Eds) Biology, Ecology, and Evolution of Gall-Inducing Arthropods.Science Publishers, Enfield (USA) & Plymouth (UK), 159–229.

[B20] Gullan PJ Williams DJ (2010) Helen May Brookes (3.11.1917–1.1.2008): her life and contributions to coccidology. Entomologia Hellenica 19: 170–172. 10.12681/eh.11587

[B21] Hardy NB Gullan PJ (2007) A new genus and four new species of felt scales on *Eucalyptus* (Hemiptera: Coccoidea: Eriococcidae) in south‐eastern Australia. Austral Entomology 46: 106–120. 10.1111/j.1440-6055.2007.00576.x

[B22] Hardy NB Gullan PJ (2010) Australian gall-inducing scale insects on *Eucalyptus*: revision of *Opisthoscelis* Schrader (Coccoidea, Eriococcidae) and descriptions of a new genus and nine new species. ZooKeys 58: 1–74. 10.3897/zookeys.58.507 PMC3088339 21594191

[B23] Hardy NB Beardsley JW Gullan PJ (2011) Uncovering diversity of Australian *Eucalyptus*‐constrained felt scales (Hemiptera: Coccoidea: Eriococcidae). Systematic Entomology 36: 497–528. 10.1111/j.1365-3113.2011.00577.x

[B24] Hill KD Johnson LAS (1995) Systematic studies in the eucalypts. 7. A revision of the bloodwoods, genus *Corymbia* (Myrtaceae). Telopea 6(2–3): 185–504. 10.7751/telopea19953017

[B25] Hodgson CJ (2002) Preliminary phylogeny of some non-margarodid Coccoidea (Hemiptera) based on adult male characters. Bollettino di Zoologia Agraria e di Bachicoltura (Milano) 33(3): 129–137.

[B26] Hoy JM (1963) A catalogue of the Eriococcidae (Homoptera: Coccoidea) of the world. New Zealand Department of Scientific and Industrial Research Bulletin 150: 1–260.

[B27] International Commission on Zoological Nomenclature (1999) International Code of Zoological Nomenclature, 4^th^ ed (with amendments). The International Trust for Zoological Nomenclature, The Natural History Museum, London, 306 pp http://www.iczn.org/code [accessed 10 September 2018]

[B28] Koteja J (1974) On the phylogeny and classification of the scale insects (Homoptera, Coccinea) (discussion based on the morphology of the mouthparts). Acta Zoologica Cracoviensia 19: 267–325.

[B29] Maskell WM (1892) Further coccid notes: with descriptions of new species, and remarks on coccids from New Zealand, Australia and elsewhere. Transactions and Proceedings of the New Zealand Institute 24(1891): 1–64.

[B30] Maskell WM (1893) Further coccid notes: with descriptions of new species from Australia, India, Sandwich Islands, Demerara, and South Pacific. Transactions and Proceedings of the New Zealand Institute 25(1892): 201–252.

[B31] Maskell WM (1896) Further coccid notes, with descriptions of new species and discussions of questions of interest. Transactions and Proceedings of the New Zealand Institute 28: 380–411.

[B32] Morrison H Morrison ER (1922) A redescription of the type species of the genera of Coccidae based on species originally described by Maskell. Proceedings of the United States National Museum (Washington) 60: 1–120. 10.5479/si.00963801.60-2407.1

[B33] Semple TL Gullan PJ Hodgson CJ Hardy NB Cook LG (2015) Systematic review of the Australian ‘bush coconut’ genus *Cystococcus* (Hemiptera: Eriococcidae) uncovers a new species from Queensland. Invertebrate Systematics 29: 287–312. 10.1071/IS14061

[B34] Taylor G Keller M (2008) Obituary. Helen May Brookes: entomologist and botanical artist. Myrmecia 44(2): 26–28.

[B35] Upton MS (1997) A Rich and Diverse Fauna: The History of the Australian National Insect Collection 1926-1991. CSIRO Publishing, Collingwood, Victoria, 386 pp.

